# The first draft genome of *Picrorhiza kurrooa*, an endangered medicinal herb from Himalayas

**DOI:** 10.1038/s41598-021-93495-z

**Published:** 2021-07-22

**Authors:** Tanvi Sharma, Nitesh Kumar Sharma, Prakash Kumar, Ganesh Panzade, Tanuja Rana, Mohit Kumar Swarnkar, Anil Kumar Singh, Dharam Singh, Ravi Shankar, Sanjay Kumar

**Affiliations:** 1 Biotechnology Division, CSIR-Institute of Himalayan Bioresource Technology (CSIR-IHBT), Palampur, Himachal Pradesh 176061 India; 2 Studio of Computational Biology and Bioinformatics, CSIR-Institute of Himalayan Bioresource Technology (CSIR-IHBT), Palampur, Himachal Pradesh 176061 India; 3 Academy of Scientific and Innovative Research (AcSIR), Ghaziabad, Uttar Pradesh 201002 India; 4 Present Address: ICAR-Indian Institute of Agricultural Biotechnology, Ranchi, 834 003 India

**Keywords:** Genomics, Sequencing, Genome informatics

## Abstract

*Picrorhiza kurrooa* is an endangered medicinal herb which is distributed across the Himalayan region at an altitude between 3000–5000 m above mean sea level. The medicinal properties of *P. kurrooa* are attributed to monoterpenoid picrosides present in leaf, rhizome and root of the plant. However, no genomic information is currently available for *P. kurrooa*, which limits our understanding about its molecular systems and associated responses. The present study brings the first assembled draft genome of *P. kurrooa* by using 227 Gb of raw data generated by Illumina and PacBio RS II sequencing platforms. The assembled genome has a size of n = ~ 1.7 Gb with 12,924 scaffolds. Four pronged assembly quality validations studies, including experimentally reported ESTs mapping and directed sequencing of the assembled contigs, confirmed high reliability of the assembly. About 76% of the genome is covered by complex repeats alone. Annotation revealed 24,798 protein coding and 9789 non-coding genes. Using the assembled genome, a total of 710 miRNAs were discovered, many of which were found responsible for molecular response against temperature changes. The miRNAs and targets were validated experimentally. The availability of draft genome sequence will aid in genetic improvement and conservation of *P. kurrooa*. Also, this study provided an efficient approach for assembling complex genomes while dealing with repeats when regular assemblers failed to progress due to repeats.

## Introduction

*Picrorhiza kurrooa* Royle ex Benth. is a perennial medicinal herb that grows in Himalayan regions of India, China, Pakistan, Bhutan and Nepal between 3000–5000 m above mean sea level. This species is a major source of bioactive constituents, mainly picrosides which are known hepatoprotective molecules^1,2^. Picrosides are iridoid glycosides present in leaf, root, and rhizome of the species^3^. *P. kurrooa* is widely harvested from natural habitat for use in traditional ayurvedic system of medicine. The species is used in a number of commercially available drug formulations like livocare, livomap, livplus and katuki for the treatment of liver ailments^4^. Recent resurgence in global demand for herbal medicines has posed additional pressure on naturally occurring populations of *P. kurrooa*^5^. Altogether, unregulated over-harvesting, lack of organized cultivation, habitat specificity and small population size have threatened the species to near extinction, making it listed as endangered species by International Union for Conservation of Nature and Natural Resources^6,7^.

Though the transcriptome of the species was deciphered by our group long back^8^, little information exists for the genome of this species. The haploid chromosome number and estimated genome size of *P. kurrooa* are 17 (2n = 34)^9^ and 1.76 Gb^10^, respectively. Whole genome sequencing is a major way to develop genomic resources and information for a species. Moreover, the advent of next generation and third generation sequencing platforms in the last few years have demonetized the genome sequencing projects, opening gateway to the book of life of several species at much faster pace. This is well reflected by the fact that now one can find projects like 1000 genome project or Earth Biogenome project. Short-read sequencing technologies have been successfully deployed to sequence homozygous haploid genomes of many plant species including *Cicer arietinum*^11^, *Cajanus cajan*^12^, *Prunus persica*^13^ and S*olanum tuberosum*^14^. However, sequencing and assembly of heterozygous plant genomes is challenging due to high amount of complex repeats in plant genomes which leads to highly fragmented repeats upon assembly. This problem is solved by having longer sequencing reads along with short sequence reads. For example, genomes of *Salvia miltiorrhiza* Bunge ^15^ and *Siraitia grosvenorii*^16^ plants were successfully assembled de novo by using hybrid assembly. To this date a total of 4758 eukaryotic genomes have been sequenced, out of which only ~ 10% are embryophyta genomes.

Keeping in view the medicinal and economical importance of *P. kurrooa,* and its endangered status, it was essential to sequence the genome that is expected to aid in improvement, better molecular interventions, and conservation of the species. Here we report the first ever draft genome sequence of *P. kurrooa* Royle ex Benth. using hybrid de novo assembly approach. Short reads from Illumina GAIIx along with long reads from PacBio RSII sequencing platform were used to reconstruct the draft sequence.

For better gene models and to identify transcriptionally active regions of the genome including transcriptionally active repeats and miRNA discovery, cDNA as well as miRNA libraries were sequenced and analyzed for the identification of differential expression pattern of miRNAs, as well as target transcripts at two temperatures. These libraries were prepared using leaf and rhizome tissue exposed to two different temperatures (15 °C and 25 °C). Since, our previous studies showed modulation of vital metabolic pathways and biological processes at 25 °C considering 15 °C as the base temperature^8^. So far, hardly any miRNA based study has been done for *P. kurrooa* due to the lack of genomic sequence. The present study has leveraged the sequenced *P. kurrooa* genome to report for the first time its miRNAs and their regulatory repertoire, opening the gateway for research towards post-transcription regulatory system of *P. kurrooa*. Besides, the draft genome also led to the discovery of transcription factors and their binding sites across the genome.

## Results and discussion

### Filtering of reads and primary de novo assembly

A total of 115 Gb sequencing read data with 67X genome coverage was obtained upon sequencing of seven genomic libraries on Illumina GAIIx sequencing platform having insert sizes ranging from 76 to 101 bp. Almost 70% reads in each read file were left after quality filtering. Out of total 115 Gb data, 80 Gb data was left for further use (Supplementary Data sheet S1).

PacBio RSII SMRT platform was used for long read sequencing. Two libraries of *P. kurrooa* with insert size 10 Kb and six libraries with 20 Kb were sequenced. A total of 255 SMRT cells were used to generate 112 Gb data (65X). A total of 24,658,090 reads were generated with minimum read length of 36 bp and maximum read length of 72,908 bp. The average read length of PacBio reads was found to be 4.57 Kb. PacBio data contained ~ 15% sequencing error in the form of indels (insertions and deletions), making it difficult to assemble. Fastq files were checked for PHRED quality, but not even a single read was found having an average QV > 13. Therefore, quality values didn’t prove to be a suitable parameter for the processing of PacBio long reads. To correct these long reads two types of strategies were used. Firstly, hybrid error correction tool proovread^17^ was used. This tool maps Illumina reads across the PacBio long reads. High quality Illumina reads help PacBio long reads to correct base pair errors of indels. After correction with proovread, only 49 Gb PacBio long read data remained. PacBio self-error correction strategy was also implemented using Canu^18^, which yielded around 41.36 Gb data. Average length of proovread corrected reads was 2.56 Kb, whereas, average read length for PacBio/Canu corrected reads was 4.85 Kb. If proovread finds a chimeric read, it splits this read into two different reads, thus, average read length of proovread corrected reads was found to be lower than PacBio self-error corrected reads. Corrected reads obtained from both the strategies were used for primary assembly draft. A total of 90.36 Gb data was used for assembly using Canu assembler with default parameters. The Canu assembler resulted into 1,32,032 contigs with average length 20 Kb, total base-pairs 2.64 Gb, and N50 value of 22.3 Kb, whereas the maximum contig length was found to be 211.563 Kb. These results were not close to the expected genome size i.e. 1.7 Gb, and the total number of contigs (1,32,032) indicated this also.

The genome assembly was also performed using DBG2OLC to compare the initial assembly statistics. A total of 735.65 Mb genome was assembled with N50 value of 20.6 Kb and 18.09 Kb average length where the maximum length of sequence was found to be 180.4 Kb. Primary assembly statistics indicated that assembled genome quality was inferior in comparison to Canu assembler. Hence, we proceeded further with the assembly obtained from Canu for further improvement with in-house developed strategy.

### Scaffolding and assembly improvement

**Figure 1:**
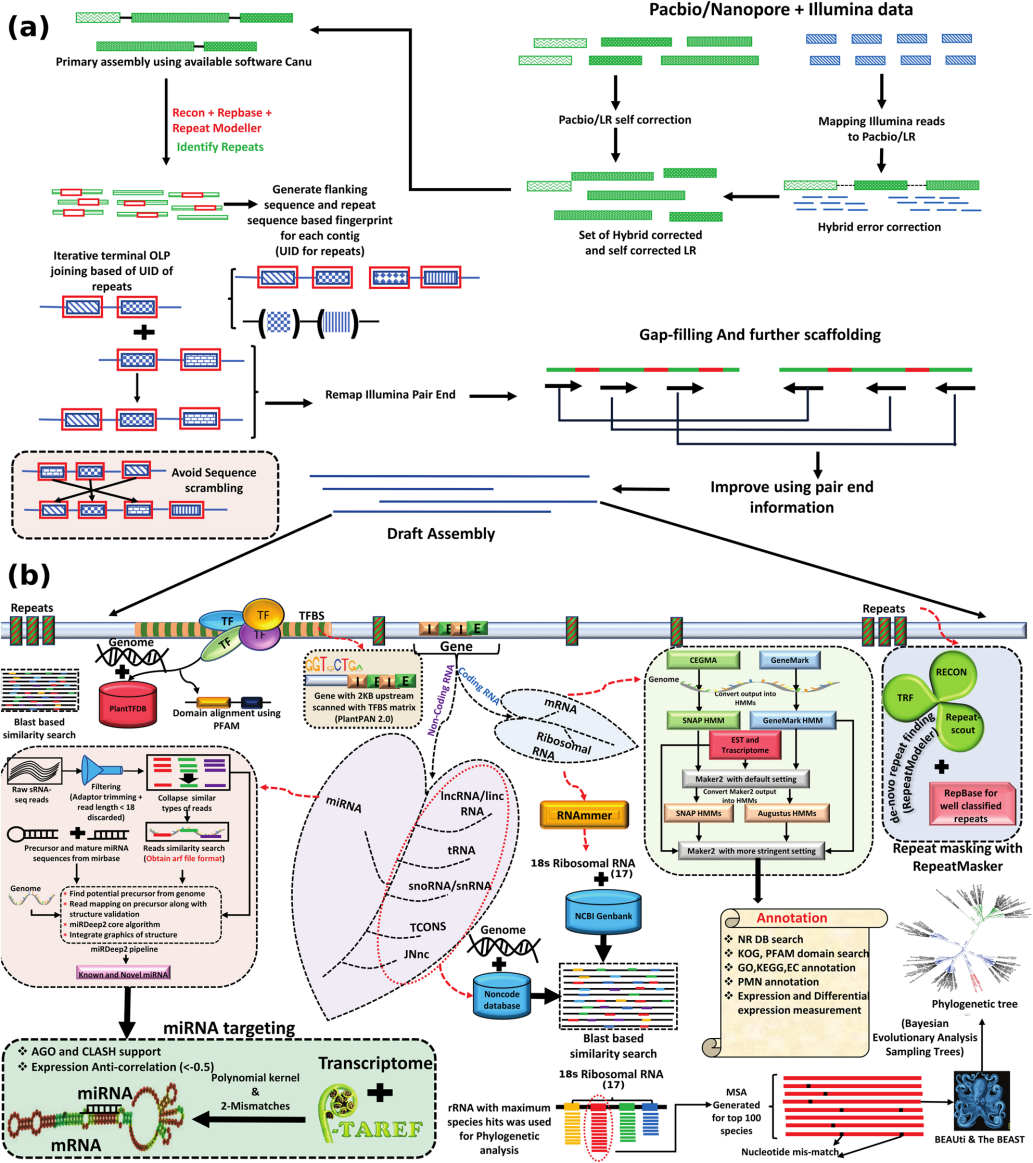
(**a**) Schematic diagram of the strategy used to join contigs into scaffolds using unique stretches of contigs. Initially PacBio long reads (LR) were corrected using self correction as well as by hybrid correction. LRs from both the strategies were used in Canu assembler for primary assembly. Primary assembly was checked for novel and known repetitive content. These contents were used to mask the assembly and generate unique tags. On the basis of unique regions and repeat families, unique identification (UID) was generated for each region. On the basis of these, UID scaffolds were generated with the help of local similarity search tool (BLAST). Further, this assembly was improved with Illumina PE read mapping. (**b**) Workflow of annotation pipeline followed for all the major components of the genome including repeats, transcription factors, genes, miRNAs, transcription factor binding sites (TFBS), phylogenetic analysis, miRNA targeting information and non-coding RNA annotation etc.

It is suggested that plant genomes are highly repetitive where general assemblers fail to perform reasonable assembly due to repeats^20^. This becomes more pronounced with the volume of complex repeats like long terminal retrotransposons (LTRs) whose single copy size may range beyond 10 Kb. Therefore, it is advisable to address the complex repeats in advance to get better assembly. For this, the repetitive elements on the primary assembly draft were discovered using RepeatModeler^21^, RepeatScout^22^, RECON^23^, Tandem Repeat Finder (TRF)^24^ and RepeatMasker^25^, combining de novo and homology based approaches. A total of 75.82% of sequenced genome appeared as repetitive. 54% of the genome contained well classified repetitive sequences, whereas, 21% were found to be unknown or novel repeats. Due to this much repetitive content usual approach like Canu failed to assemble the genome any further. Therefore, a novel strategy was used for further improved assembly and scaffolding (Fig. 1a). The repeat annotated genome was studied for the unique regions where even repeat copies were uniquely identified and in contextual manner. After masking all the contigs, those contigs which contained at least 100 bp unique unmasked stretch were separated from those which did not contain even a single unique stretch of 100 bp. There were 22,264 (280 Mb) contigs with complete repeats, whereas, 1,09,768 contigs (2.31 Gb) were found to have 625 Mb unique regions. BLASTN was applied to find similarities between these local unique regions of contigs. Some important criteria, as discussed below, were used to find the best match for any given contig.

First, sequential appearance of the unique regions must be similar to another contig, which was considered as the best hit. It prevented scrambling of unique regions. To consider one unique region of one contig similar to another unique region of another contig, minimum 80% similarity was required. The second criterion for selection was that unique regions of a contig, matching with another contig must be at least 500 bp long. This ensured that occurrence of a random 500 bp long stretch by chance would be quite low. The third criterion was that the orientation of unique stretches must be the same in both the contigs. The fourth criterion was that if a contig showed more than one probable merging candidates, the participating partner sequences were verified for similarity to form consensus based merging. The fifth criteria was to check the repeat family similarity and proportion in between unique locus of contigs. If a contig was found showing probable merging partners with another contig on the basis of unique regions, then repeat families in between those unique regions must agree with this merging. Similarity between repeats was dependent only on family based similarity. Considering these five criteria, this process was repeated iteratively till no further merger of contigs happend. After 16 cycles, this process reached at its saturation stage as significant similarity among contigs disappeared (Supplementary Fig S1). After completion of this process, contig numbers came down from 1,09,768 contigs to 12,924 contigs, maximum length of contig increased from 2,11,563 bp (211.5 Kb) to 6,38,644 bp (638.6 Kb). N50 value of assembly increased from 22.3 to 129.6 Kb. Total number of base pairs came down to 1.57 Gb from 2.31 Gb (Table 1). This showcased the efficiency of the applied approach in providing assembling solution, when regular assemblers fail to perform better due to complex repeats. The applied strategy might be used in dealing with such complex and big genomes. Considering it as the final draft assembly, different types of annotation strategies were used for genome annotation (Fig. 1b).

**Table 1:** Statistics of draft genome assembly of *P. kurrooa.*

Total contigs	12,924
Maximum contig Length	6,38,644 (638 Kb)
Contig Length > 1000 bp	100%
N50	1,29,641 (129.6 Kb)
Total base	1,57,22,32,662 (1.57 Gb)
Total average	1,21,652.17 (121 Kb)
GC Percentage	37.89

**Figure 2:**
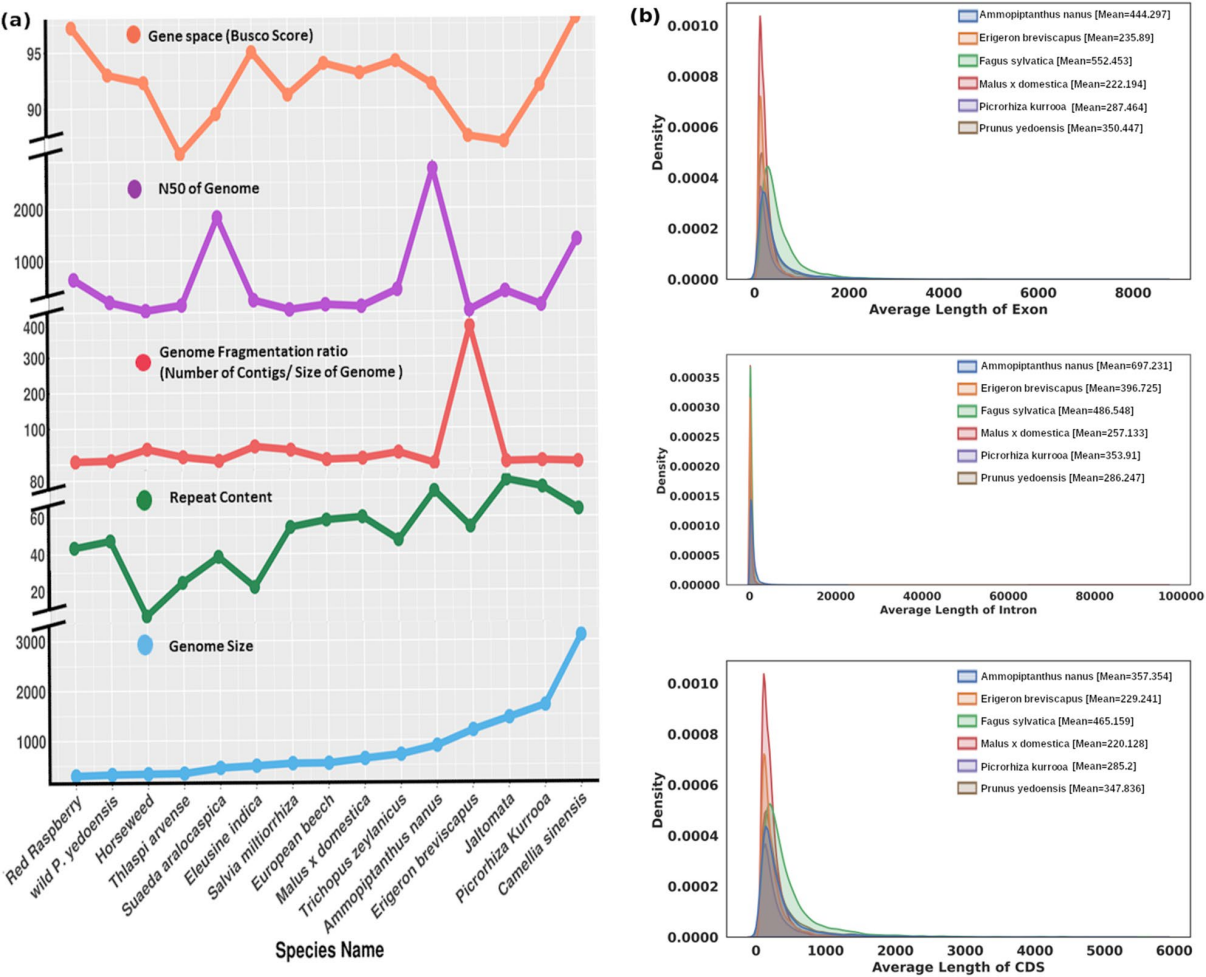
(**a**) Comparative plot of species depicting the genome size relationship with repeat content. With increasing size of genome, assembly fragmentation ratio also increases. N50 of *P. kurrooa* genome was higher than the previously reported assemblies and overall BUSCO score was 92.3% for the conserved genes. (**b**) Comparative plot of length distribution of exon, intron and coding sequence (CDS) regions of six different species along with *P. kurrooa*.

For primary assembly draft, this might be considered satisfactory. The N50 values of previously published primary genome assembly drafts for *Oryza sativa*^26^, *Cannabis sativa*^27^, *Phoenix dactylifera*^28^ and *Utricularia gibba*^29^ had N50 value of 12 Kb, 16 Kb, 30 Kb, and 95 Kb respectively. *P. kurrooa’s* assembly was compared with a few published primary assembly draft of some species^30–43^ (Fig. 2a), in which hybrid approach using long and short read sequencing was used. Interestingly, these published genomes also used paired-end and mate-pair libraries from Illumina and 10-20 Kbs library from PacBio. In most of the studies where mate-pair libraries were used, SOAPdenovo2^44^ assembler was preferred for primary assembly which was followed by primary assembly polishing using PacBio reads with PBJelly^45^. MaSuRCA^46^, DBG2OLC^19^ and Platanus^47^ assemblers were preferred in case of absence of mate-pairs libraries. It was found that genome size was proportional to the repeat content. Therefore, it becomes challenging to assemble a genome with increase in its size, as the repeat share increases. *Rubus idaeus*, *Prunus yedoensis*, *Thlaspi arvense*, *Conyza canadensis*, *Suaeda aralocaspica*, and *Eleusine indica* are some species which had a genome size < 500 Mb and the repeat content was < 50%. Whereas, *Ammopiptanthus nanus*, *Erigeron breviscapus*, *Jaltomata sinuosa*, *P. kurrooa* and *Camellia sinensis* have a genome size of > 800 Mb and the repeat content was > 50%. This comparison suggested that repeat content is a huge hurdle for scaffolding/assembly. Therefore, when repeat content is high, assembly is more fragmented. Long read assemblers like Canu take this problem into consideration but these tools are dependent on k-mer searching based approach for repeat finding which is suitable only for simple repeats. In our case Canu reported only 8% repeats in overall assembly and gave a highly fragmented assembly. Also, when the genome size was compared with the number of contigs reported in previous studies, it was found that as the genome size increases, number of contigs also increases or assemblers failed to further resolve the assembly. Ratio of number of contigs versus genome size showed that *Rubus idaeus* had a genome size of 299.94 Mb with a total of 2145 contigs, making the ratio 7.15. In our case where genome was almost six times (1.7 Gb) of *Rubus idaeus*, number of contigs were 12,924 with a similar ratio of 7.60. The ratio suggested that lower the ratio, less fragmented would be the assembly. The ratio was proportional to the genome size, shorter the genome, lesser the fragmentation and contigs number. Hence, the assembly achieved for *P. kurrooa* was better for the given genome size. N50 value for *P. kurrooa*’s (129.6 Kb) assembly was higher as compared to assemblies of shorter genomes like *Conyza canadensis* (33.56 Kb), *Salvia miltiorrhiza* (51.02 Kb), *Malus x domestica *(111.6 Kb) and *Erigeron breviscapus *(31.5 Kb). Similarly, gene space covered by *P. kurrooa*’s (92.3%) assembly was higher than many species compared here. Therefore, the applied repeat handling approach ensured better genome assembly (Fig. 2a).

Total GC content in the assembled genome was 37.89% with 44.95% GC content in coding sequences which was well within range of genomic and coding GC content in dicots species. Genomic DNA containing high GC content were reported to be more thermostable^48^.

### Four different levels of assembly validation

To further evaluate the completeness of assembly, four different levels of validation were performed. For the first level, the genome sequence was evaluated by BUSCO^49^. BUSCO assesses the genome assembly for its completeness using single copy orthologs which are expected to be conserved during evolution and therefore, these are expected to be conserved in genome as well. The analysis reported that 94.2% BUSCO virdiplantae genes were covered in the assembled *P. kurrooa* genome.

For second level of validation, 500 published and experimentally validated *P. kurrooa* ESTs were searched against the assembled genome. These ESTs were also used for transcriptome validation in one of our previous studies on transcriptome of *P. kurrooa*. A total of 414 significant hits were found for EST dataset with E-value < 1e−05, while retaining 95.19% average identity. These 414 ESTs showed correct alignment in continuous form on genome that suggested good quality of genome assembly. In a previous study on *Arabidopsis thaliana,* around 87% of ESTs could be aligned on the predicted gene set, while this number was quite low (64%) for human dataset. Compared to this, the *P. kurrooa* genome assembly scored far better.

At the third level of validation, PLAZA^50^ core gene families (coreGF) were used. These families are conserved among plant species with a predefined lineage. 55 species are integrated with structural and functional annotation in PLAZA 4.0. *P. kurrooa* was categorized into Lamiales category in predefined lineage of these 55 species. For Lamiales category, two species *Erythranthe guttata* and *Utricularia gibba* are included in this database. There are 19,050 unique orthologous families reported in this database for Lamiales. These orthologous families were searched across the *P. kurrooa*’s genome and 14,212 (74.6%) orthologous families were found with significant hits. Thereafter, the common orthologous families between *Erythranthe guttata* and *Utricularia gibba* were checked which are reported within this database itself. It was found that *Erythranthe guttata* has a total of 12,226 orthologous families while *Utricularia gibba* has a total of 14,772 orthologous families. In between these two, there are 7948 (65%) common orthologous groups, which is quite less than *P. kurrooa’s* figures (74.6%).

Finally, 11 supra contigs of assembled *P. kurrooa* genome were randomly selected for experimental validation. All the primers designed from 5′ and 3′ ends of eleven supracontigs gave expected amplicon size after PCR at the standardized annealing temperatures (Supplementary Fig. S3; Supplementary Table S1). The sequences of the eluted amplicons showed 100% sequence identity to the sequences of *P. kurrooa* contigs targeted for PCR amplification.

### Repetitive elements in the genome

Plant genomes contain much smaller coding regions as compared to repeats. The repetitive elements may cover upto 90–95% of the genome^51^. Repetitive elements may be considered central to almost every genomic subject and issues, from assembling challenges to chromatin state determination. Repetitive elements play a major role in the evolution and stand as an integral component for genomic stability and regulatory repertoire^52^. Therefore, to investigate total repetitive content in *P. kurrooa*, RepeatModeler, RepeatScout, RECON, Tandem Repeat Finder (TRF) and RepeatMasker were run across the draft genome. This included de novo as well as well-characterized repeats in RepBase database. A total of 76.06% interspersed repeats were found in the draft assembly (Supplementary Table S2).

**Figure 3:**
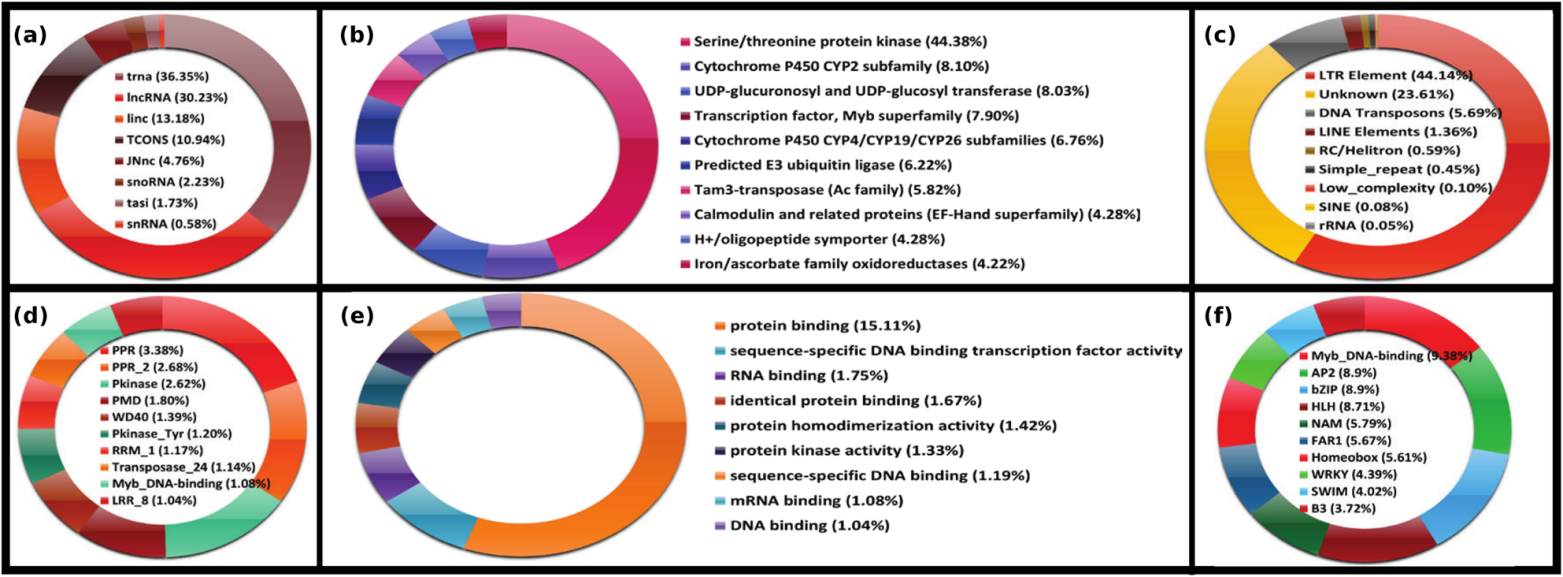
Top 10 categories from six different types of annotations (**a**) non-coding RNAs, (**b**) KOG, (**c**) repeats, (**d**) PFAM domain, (**e**) PFAM functional classification, and (**f**) transcription factors.

In the draft assembly, 23.61% were unclassified repeats and 52.45% were well-classified repeats. A total of 42% repeats emerged from Gypsy and Copia long terminal retrotransposons (LTR elements) repeats categories alone (Fig. 3c). Transposable elements (TEs) are considered as major constituent for genome evolution and make significant fraction of plant genomes. Comparative study with other plant genomes depicted that plant repeat content increases with increasing genome size. Genome size of *Arabidopsis thaliana* is 125 Mb and it contains 14% repeats, *Oryza sativa* with genome size of 430 Mb contains 26% repetitive genome, whereas, plants like *Zea mays* (2.3 Gb), *Glycine max* (1.11 Gb), and *Triticum aestivum* (17 Gb), which have higher genome size, have 85%, 57%, and 80% repeat content, respectively. The draft assembly of *P. kurrooa* also followed the same trend with ~ 76% repeat content, reflecting the characterstic of a big genome.

The draft assembly contained 129.21 Mb (8.24%) tandem repeats. MISA^53^ micro-satellite finder was also used to find simple tandem repeats in assembled genome, which identified 2,58,408 simple sequence repeats in genome, out of which, 66.29% were single nucleotide repeats, 22.94% were di-nucleotide repeats and 9.68% were tri-nucleotide repeats (Supplementary Table S3). Total 0.278% of the genome belonged to this category.

### Protein coding genes annotation

Using CEGMA^54^, SNAP^55^, GeneMark^56^, AUGUSTUS^57^and Maker2^58^ pipelines as described in the methods section, a total of 24,798 potential protein coding unique genic regions were found in the primary assembly draft, which included multicopy genes also (making the total count of 51,185 protein coding regions). This covered 116.25 Mb of genome, which is around 6.8% of the whole genome. The length of these genes ranged from 135 to 24 Kb. 10,683 genes had two or more number of copies, whereas 14,115 genes had a single copy number displaying typical property of plant genomes which are usually rich in multi-copy genes. Number of copies ranged from 2 to 29, which suggested that these genes were required very frequently by the plant. In order to investigate the type of genes present in high copy number, top 100 genes with maximum number of copies were analyzed. Most of these genes fell either into hypothetical protein category or in ribosomal protein category. Some of the genes belonged to eukaryotic translation initiation factor 4G (eIF4G) or transcription factors category containing zinc finger domain. Previous study on *Brassica rapa* suggested that multiple copies of eukaryotic translation initiation factor 4E (eIF4E) enabled redundant plant translational machinery, resulting in diversification and emergence of resistance.

In order to assess the completeness of the genic regions in the assembled draft genome, the length distribution of exon, intron, and coding sequence (CDS) regions of the genes was compared with the published genomes for five different species including *Ammopiptanthus nanus*, *Erigeron breviscapus*, *Fagus sylvatica*, *Malus x domestica*, *Prunus yedoensis*. Average exon, intron and CDS length of *P. kurrooa* was found to be 287.464 bases, 353.91 bases and 285.2 bases, respectively. The distribution of all these values were found in the similar range as was observed for the five compared species, suggesting comparable assembly of the genome with almost similar level of information on genes (Fig. 2b). This was expected as unique regions of genomes hardly cause poor recognition of partners during assembling and achieve smoother assembling. Fragmentation occurs due to poor assembling partner recognition which is usually posed by complex repeat regions. Out of 24,798 unigenes, 17,773 belonged to 2973 different PFAM categories. A total of 27,910 PFAM HMMs were found in 17,773 unigenes. Among these, 48.98%, 40.59%, 9.7% belonged to PFAM domain, PFAM family and PFAM repeats, respectively. Pentatricopeptide repeat (3.38), PPR repeat family (2.68), protein kinase (2.62), plant mobile domain (1.80), WD40 or β-transducin repeat (1.39), tyrosine kinase (1.20), RNA recognition motif (1.17), plant transposase (1.14), Myb_DNA-binding (1.08), leucine-rich repeat (1.04) were among the top ten most abundant family in PFAM analysis (Fig. 3d). Out of 24,798 unigenes, 13,506 belonged to Eukaryotic Orthologous Groups (KOG). Serine/threonine protein kinase (signal transduction mechanisms), cytochrome P450 CYP2 subfamily (secondary metabolites biosynthesis, transport and catabolism), UDP-glucuronosyl and UDP-glucosyl transferase (carbohydrate transport and metabolism, energy production and conversion), transcription factor, Myb superfamily (transcription), cytochrome P450 CYP4/CYP19/CYP26 subfamilies (secondary metabolites biosynthesis, transport and catabolism, lipid transport and metabolism), predicted E3 ubiquitin ligase (post-translational modification, protein turnover, chaperones), tam3-transposase (replication, recombination and repair), calmodulin and related proteins (EF-Hand superfamily) (signal transduction mechanisms), H^+^/oligopeptide symporter (amino acid transport and metabolism), iron/ascorbate family oxidoreductases (secondary metabolites biosynthesis, transport and catabolism, general function prediction only) were among the most abundant orthologous groups (Fig. 3b). Similar analysis was done separately for those genes which had more than one copy. Out of 37,072 genes, 15.11% belonged to protein binding category. Similarly, 2.57% genes belonged to sequence specific DNA binding transcription factor activity, 1.75% belonged to RNA binding, 1.67% belonged to protein binding related function (Fig. 3e). In PFAM analysis 7778 genes had 2254 unique PFAM domains. Due to multiple copies of genes, 41,966 PFAM domain copies were found to exist in these genes. These copies followed the same pattern of abundance as overall genes analysis. Maximum number of copies were found for pentatricopeptide repeat, PPR repeat family, protein kinase, plant mobile domain, WD40 or β-transducin repeat. 5871 genes were found clustered with at least one orthologous group. Similar to PFAM analysis, KOG analysis for multi-copy genes also followed same pattern of abundance for all genes in terms of most abundant terms.

A total of 1612 unigenes were found to be putative transcription factors on the basis of two different annotation approaches. In the first approach the annotations from BLAST/ Annot8r^59^ were considered as transcription factor based on significance score and homology against the known transcription factors. A total of 1157 transcription factors were found by this approach. Besides this, transcription factors were also searched on the basis of PFAM domain search. A total of 829 unigenes with transcription factor domain were recorded. When the data from these two analyses were merged and unified, a total 1612 unigenes appeared as transcription factors. All of these transcripts were searched against Planttfdb^60^. Out of these 1612 putative transcription factors, MYB (9.38%), AP2 (8.9%), bZIP (8.9%) and HLH (8.71%) transcription factor families were found to be the most abundant (Fig. 3f). Along with transcription factor characterization, their binding sites were also scanned across the genome. A total of 981 different transcription factors belonging to 82 different plant transcription families with a total of 1881 transcription factor binding sites (TFBS) matrix models were download from PlantPAN2.0^61^ database. These matrices were scanned across the genes along with their 2 Kb upstream regions. A total of 1,48,38,54,421 TFBS were found which appeared very high. To filter out significant binding sites, matrices were mapped to their respective TF family. Transcription factors, belonging to those families and genes on which their binding sites were found, were further studied for expression correlation >  = 0.8 with significant p-value (< 0.05). A total of 6,23,434 binding sites belonged to 1154 TFs containing 35 TF families. The most abundant binding sites were found for MYB (14%), bZIP (12.9%) and AP2 (11.4%) followed by Homeobox and HLH.

### Major non-coding elements

Those transcripts which do not serve as template for functional protein synthesis are called as non-coding RNAs (ncRNAs). Some of the recent studies indicated that under abiotic stress, plants produce several hundreds of non-polyadenylated lncRNAs^62^. Dynamic regulations of lncRNAs act as regulating factor in development and stress response. To find non-coding elements in the draft genome assembly, plant NONCODE database^63^ was used. Using BLASTN, different non-coding elements were located in the genome against NONCODE database. A total of 9789 non-coding elements, including lncRNA, tasiRNA, tRNA, and snoRNA were found in our data. Among 9789 non-coding elements, 3556 (36.35%) belonged to tRNAs, 2957 belonged to lncRNA (30.23%), 1289 belonged to lincRNAs (13.18%) and 1070 (10.94%) belonged to TCONS (novel type of lincRNAs) (Fig. 3a). This must be noted that plant non-coding RNAs are not well annotated as in animals, and lag behind in methods and software to identify them in plants.

### Phylogenetic analysis of *P. kurrooa*

Different species have evolved with time through natural selection. Underlying mechanism of this evolution is genomic mutation which occur spontaneously while following molecular clock. *P. kurrooa* is listed as an endangered plant species due to uncontrolled harvesting, making it essential for evolutionary analysis^64^. Out of 24,798 unigene transcripts BLAST searched against NR database, 24,517 returned successful hits. Out of these hits, maximum hits were found against *Sesamum indicum* (36.30%) followed by for *Handroanthus impetiginosus* (18.13%), *Erythranthe guttata* (8.91%) and *Olea europaea *var*. sylvestris* (4.82%) (Supplementary Table S4). These four species covered 68% of the total hits. *Sesamum indicum* belongs to Pedaliaceae family, but *P. kurrooa* does not belong to this family, yet *P. kurrooa* follows same taxonomy till the order level. Since the genome of *Sesamum indicum* is well annotated, *P. kurrooa* might have shown maximum similarity with this species based on abundance of transcript data. Out of the top 10 most abundant species, none of them belonged to *P*. *kurrooa* family or genus. *P*. *kurrooa* belongs to family Plantaginaceae and NR database consisted a very limited number of proteins belonging to this family. Due to lack of any well annotated genome in *Picrorhiza*’*s* genus, it showed the similarity with *Sesamum indicum* at order level, as both species share the same order, Lamiales.

**Figure 4:**
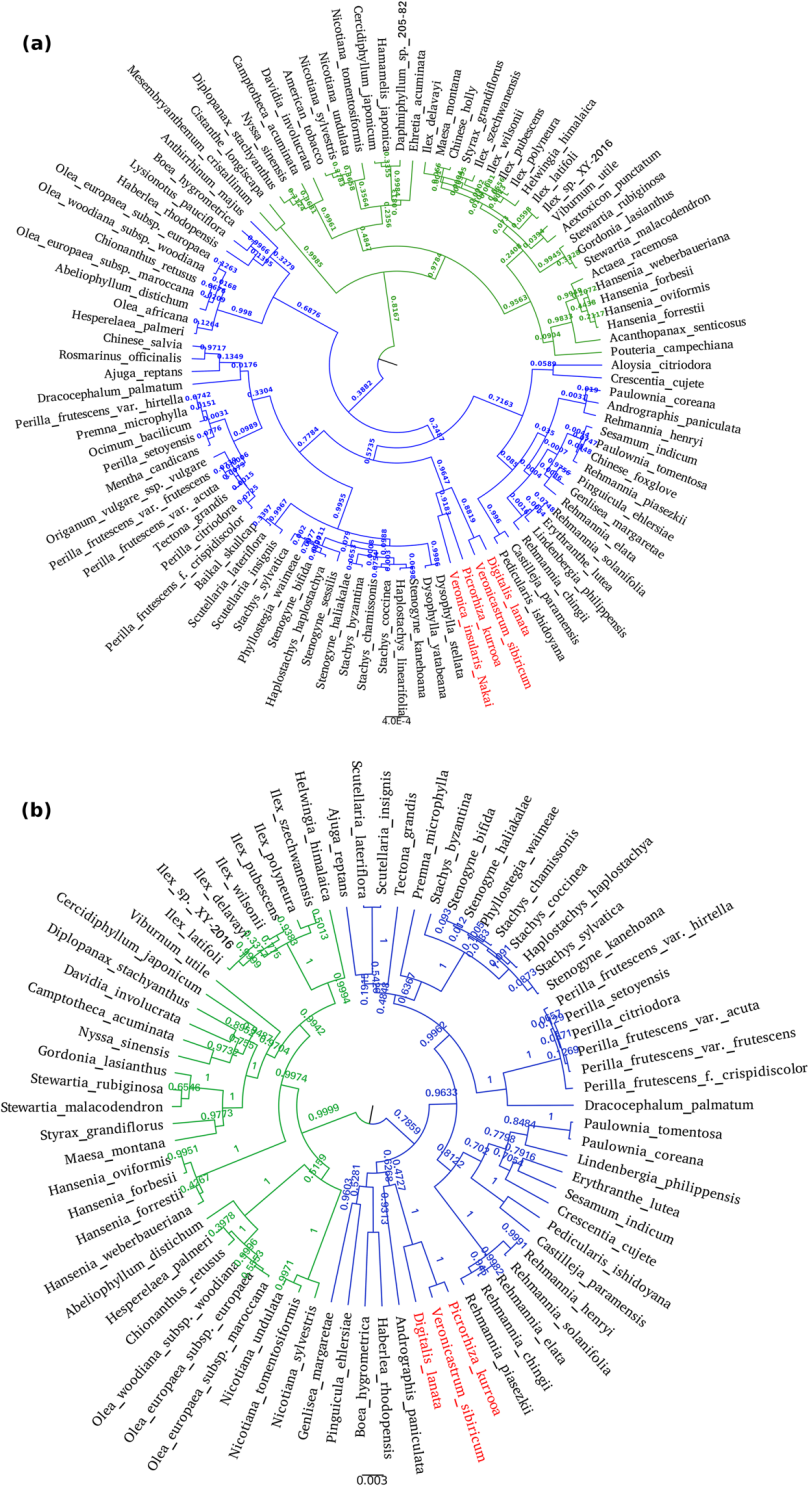
(**a**) Phylogenetic tree on the basis of ribosomal RNA, showing 100 different taxons on the nodes and posterior probability onto branches. Two major clusters with blue and green colors on branches are showing major classification of Lamiales (blue color), other orders are depicted in green color. Red color taxon represents small cluster of *P. kurrooa* with other members of Veroniceae tribe along with *Digitalis lanata.* (**b**) Phylogenetic tree with 74 species obtained on the basis of a chloroplast gene. This tree was found strongly in concordance with the one obtained through ribosomal RNA analysis.

Ribosomal RNAs were identified in *P. kurrooa* draft genome using RNAmmer^65^. A total of 17 unique 18S ribosomal RNAs were found in the data. Each ribosomal RNA had multiple copies. Maximum number of copies for a ribosomal RNA was 891, whereas, minimum number was 136 copies. Further, these 17 ribosomal RNAs were searched using BLASTN in NCBI's nucleotide database. Each ribosomal RNA returned multiple hits among different species. For phylogenetic analysis, single 18S ribosomal RNA was selected. Cross species sequences were aligned using clustalW. Alignments were loaded for phylogenetic analysis into BEASTv1.10.4^66^ with strict molecular clock and GTR substitution model for tree annotation. Generated tree was loaded into figtree for visualization. Phylogenetic tree clearly distinguished *P. kurrooa* among all other taxon which were not close to this species taxonomically (Fig. 4a). On other hand, it also placed *P. kurrooa* with *Veronica insularis *Nakai*.* These both species share same tribe, *Veroniceae*. However, *Digitalis lanata* was also clustered into *P. kurrooa* group. Reason behind that could be that this species shares family with Veroniceae trib*e* and any other tribe did not have major similarity with this group in our selected species. The major cluster was also able to distinguish Lamiales order with all the other order in the tree. In a similar manner, all other small clusters clearly reflected the common taxonomical relationship among the members. For instance, Solanales cluster had all the *Nicotiana*’s family members. Apiales were also distinguishable in the tree as a single cluster.

The top 100 closest species were further considered for another phylogenetic analysis on the basis of conserved genes and chloroplast genes. However, out of 100 species, genome sequence was available for only nine species and transcriptome was available for thirteen species only. Hence, several conserved house-keeping gene sequences were not available across all the considered 100 species. Maximum overlap of any conserved gene across these species was found only for 39 species. Therefore, chloroplast genes were preferred over conserved house-keeping genes. Nucleotide sequence of photosystem II protein D1 (chloroplast) was considered for phylogenetic analysis. This gene sequence was available for 74 species. The phylogenetic analysis with chloroplast gene also classified *P. kurrooa* in Veroniceae tribe. Similar to the 18S ribosomal RNA analysis, here also Lamiales order was distinguishable from all the other order in analysis. The clusters formed here also showed high similarity with those obtained with 18S ribosomal RNA based analysis (Fig. 4b).

Another phylogenetic analysis was performed using AAI-profiler, an amino acids indexer for identity^67^. It is a proteome based homology search tool. From the draft assembly the proteome was created using MAKER2 program on the basis of six frames ORF. This proteome data for 24,798 transcripts was submitted to AAI. Scatterplot shows AAI percentage on x axis whereas matched fraction is plotted on y-axis. Each data point represents a different species. Color coding of data point tells us about the order of eukaryotes. If a data point is given at the top right corner of this plot it indicates that species is completely matched with database. At top right corner AAI value and matched fraction both will be 1. In our data this was not the case, as this is a novel genome assembly. In our data two subclasses, asterids and rosids showed the maximum abundance (Supplementary Fig. S4a). Both subclasses belonged to Pentapetalae clade. Top most species with maximum average amino acid identity of 0.723 and matched fraction of 0.457 was *Nicotiana tabacum*. *Nicotiana tabacum* belongs to asterids subclass, which was also present in the rRNA based phylogenetic analysis. However, it was present in a different group in phylogenetic tree, though the two groups consisting of 6 species (*Angelica gigas*, *Asiatic ginseng*, *Hesperelaea palmeri*, *P. kurrooa, American tobacco* and *Antirrhinum majus*) clustered together with 100% clade credibility value. Next top species in AAI profiler analysis was *Populus trichocarpa* with average amino acid identity of 0.702 with matched fraction of 0.444. It belongs to rosid subclass.

Among the the top 20 species in AAI profiler, 12 species belonged to subclass rosid and 8 belonged to subclass asterid. *P. kurrooa*'s order Lamiales comes under asterid subclass. Out of 20 species in AAI profiler analysis, only two species including *Handroanthus impetiginosus* and *Erythranthe guttata* share same order Lamiales with *P. kurrooa.* Krona plot of *P. kurrooa* showed almost similar result to what was found from BLAST result of transcripts against NR database, as in this plot each query protein was assigned to the species of closest match (ranking by alignment bit score) (Supplementary Fig. S4b). As high as 57% of proteins were assigned to two species, *Handroanthus impetiginosus* (38%) and *Erythranthe guttata* (19%).

With more than 23,000 species, Lamiales is considered as one of the largest order of flowering plants. Genus *Picrorhiza* contains only one species in “Genera Plantarum”*.* This genus is classified into Lamiales order and four different analysis also suggested the same. All of the four analyses clustered *P. kurrooa* into the Lamiales order with sufficient confidence. In transcript based analysis top three species belonged to Lamiales order. In ribosomal RNA based analyses also *P. kurrooa* was clustered into Lamiales group along with Veroniceae trib*e*’s species*.* Similar to ribosomal RNA based analysis, chloroplast gene based analysis also classified *P. kurrooa* into Veroniceae tribe. Amino acid identity based analysis also showed the maximum AAI with asterid subclass which is super class of Lamiales. Among all four analyses, rRNA and chloroplast gene based analysis may be considered most significant due to conservative nature of ribosomal RNAs and chloroplast genes.

### Transcriptome analysis for annotated genes at two temperatures (15 °C and 25 °C) in leaf and rhizome tissue

De novo transcriptome sequencing of *P. kurrooa* leaves at 15 °C and 25 °C by our group had revealed enhanced accumulation of picrosides at 15 °C with concomitant increase in expression of key genes of the biosynthetic pathway^8^. Since leaves and rhizomes are the main sites for picroside biosynthesis, an understanding of gene expression in tissue specific manner was central to identify the key genes and regulators of complex biological pathways. Besides this, transcriptome data helps in building better gene models. Transcriptome sequencing of these tissues was performed on Illumina sequencing platform. After initial filtering of the read data, it was mapped to assembled transcripts models of the genome obtained through MAKER2. Counts of mapped number of reads under different conditions were normalized into FPKM to find out expression of the transcripts. Differentially expressed transcripts were identified using EdgeR tool with p-value < = 0.05. To functionally annotate, transcript search was performed against Uniprot database. Top hits for each query were searched for associated Gene Ontology (GO), Kyoto Encyclopedia of Genes and Genomes (KEGG) and Enzyme Commission Codes (EC). Significant GO annotation were found for 20,559 out of 24,798 transcripts. These transcripts were further categorized into biological process and molecular function classes (Supplementary Data sheet S2). Biological process category showed that meristem maintenance, embryo development ending in seed dormancy, response to abscisic acid stimulus, response to salt stress, meristem development, response to cold and regulation of transcription were under highly represented groups which showed that plant was responsive to cold while maintaining its growth (Supplementary Fig. S5). Protein binding, sequence-specific DNA binding, transcription factor activity, identical protein binding, RNA binding, protein kinase activity, protein homodimerization activity, DNA binding, sequence-specific DNA binding and mRNA binding were under the highest over-represented molecular function categories (Supplementary Fig. S5). EC classification was found for 12,192 transcripts, whereas KEGG classification was found for 12,091 transcripts. Out of 12,192 transcripts with EC hits, maximum number of transcripts belonged to the serine/threonine protein kinase enzyme class (18.84%). Similarly, most abundant KEGG pathways were plant hormone signal transduction, plant-pathogen interaction, ribosome, RNA transport, spliceosome, protein processing in endoplasmic reticulum, starch and sucrose metabolism, tight junction and endocytosis pathways (Supplementary Fig. S5).

To understand the temperature mediated gene expression, differentially expressed transcripts for two tissues were compared at 15 °C and 25 °C (Supplementary Data sheet S3–6). In biological process category, transcripts associated with response to heat, response to karrikin, defense response to fungus, response to abscisic acid stimulus, and response to wound healing were most abundant at 25 °C in both the tissues (Supplementary Fig. S6a; S6c). Overall upregulation of stress responsive transcripts at 25 °C suggested plant to be under stress. In leaf tissue, transcripts related to photosynthesis, lignin biosynthetic process, carotenoid biosynthetic process, chlorophyll catabolic process, and chloroplast organization were most abundant and were downregulated at 25 °C (Supplementary Fig. S6c).

Plant metabolic network (PMN) is a database which contains data for over 350 plant species metabolic network information. Transcripts from our analysis were also searched against PMN database. It was found that 3980 unigenes were present in PMN. Their transcripts were directly mapped onto metabolic network and pathways. Highest number of transcripts belonged to 26,27-dehydrozymosterol metabolism pathways, C4 photosynthetic carbon assimilation cycle, matairesinol biosynthesis, justicidin B biosynthesis, sesamin biosynthesis, gluconeogenesis I, homogalacturonan degradation, glycolysis I and gluconeogenesis III (Supplementary Fig. S5). Transcripts belonging to ajmaline and sarpagine biosynthesis, glutathione-mediated detoxification II and flavonoid biosynthesis pathways were upregulated at 25 °C in both the tissues (Supplementary Fig. S6b; S6d). Upregulation of flavonoid pathway genes at 25 °C in both the tissues suggested their role in acclimation to temperature stress. Downregulated transcripts in rhizome tissue at 25 °C were mostly falling into cellulose biosynthesis, suberin monomers biosynthesis, 26,27-dehydrozymosterol metabolism, matairesinol biosynthesis, phenylpropanoids methylation, starch biosynthesis, and sucrose degradation III (sucrose invertase) (Supplementary Fig. S6b). Downregulated transcripts in leaf tissue exposed to 25 °C belonged to Calvin–Benson–Bassham cycle, photosynthesis light reactions, pyridoxal 5′-phosphate biosynthesis II, gluconeogenesis I, gluconeogenesis III, glycolysis I (from glucose 6-phosphate), glycolysis II (from fructose 6-phosphate), sucrose biosynthesis I (Supplementary Fig. S6d). Downregulation of photosynthesis related pathways at high temperature suggested plant to be under stress at 25 °C.

For differentially expressed genes, top 30 unigenes were selected on the basis of logFC for each condition (Supplementary Fig. S2) and heatmaps were prepared. Under each condition only those genes were selected which had (RPKM > = 0.5). EdgeR generally shows maximum fold change for those transcripts which do not have any expression at all in one condition and a significant expression in another condition. Heatmaps depicted that several transcripts belonging to some important biological category such as response to abscisic acid stimulus, defense response to fungus and defense response to bacterium showed major upregulated fold change in rhizome exposed to 25 °C. Likewise, transcripts related to defense response to fungus, systemic acquired resistance, and response to phenylpropanoid were upregulated in leaf tissue exposed to 25 °C. Transcripts belonging to cell growth, monosaccharide transport and regulation of transcription were upregulated in rhizome tissue exposed to 15 °C. Light dependent chlorophyll biosynthetic process, photosynthesis and carbohydrate metabolism related transcripts showed upregulation at 15 °C in leaf tissue.

### miRNAs and their target analysis at two temperatures (15 °C and 25 °C) in leaf and rhizome tissue

miRNAs are key regulators of gene expression at post transcriptional level that control translation or stability of target mRNAs. Numerous studies reported a role of miRNA’s in coping up with environmental stresses in plants. Environmental stresses not only lead to aberrant expression of miRNAs but also induce synthesis of new miRNAs^68^. Two tissues (leaf and rhizome) at two temperatures (15 °C and 25 °C) of *P. kurrooa* were studied for understanding miRNA mediated gene regulation. Small RNA libraries from leaf and rhizome tissues were sequenced. This is the first small-RNAseq study done ever for *P. kurrooa*. A total of 1,87,89,667 and 2,97,82,716 sRNA reads were generated from leaf tissue exposed to 15 °C and 25 °C (L15 and L25), respectively, while 2,41,20,010 and 22,61,141 reads were generated at 15 °C and 25 °C (R15 and R25), respectively, for rhizome tissue. After quality filtering and adaptor removing 1,80,22,962 reads were obtained. Remaining reads were clustered together and mapped across the newly assembled draft genome using Bowtie. Due to mismatch limitations in the seed of Bowtie mapping algorithm, short sRNA reads mapped poorly across PacBio obtained reference genome. Therefore, reads having length between 18 to 25 bp were selected and mapped across the assembled genome using BLAST. BLAST hits with up to 5 mismatches were allowed, and reads with full length mapping were considered as mapped one. Out of 21,474 unique reads, 20,362 reads were mapped to the genome. A total of 710 miRNAs were identified in the draft assembly of *P. kurrooa*. Out of these 710 miRNAs, 167 miRNAs were novel miRNAs which were not reported in miRBase, whereas 543 known miRNA were present across different species and reported at miRBase (Supplementary Data sheet S7).

A total of 72,35,625 interactions were found between miRNAs and their targets with strong anti-correlation for expression. These interactions were covering 654 miRNAs and 24,384 targeted transcripts. Out of these 654 miRNAs, 151 were novel miRNAs whereas 503 miRNAs were present in miRBase. Out of these miRNAs, pkr-miR4372a had the maximum number of target genes (14,279) whereas pkr-miR-BART7-5p had the minimum number of targets genes (1890). Hypothetical protein MIMGU_mgv1a015711mg had the maximum number of targeting miRNAs (524). Almost similar number (523 and 522) of miRNAs were targeting zinc finger BED domain-containing protein and methyl-CpG-binding domain-containing proteins, respectively. Both proteins play an important role in transcription control, suggesting high degree of control on them. There were 271 interactions supported by degradome data covering 35 miRNAs (3 novel miRNAs) and 231 genes, while 9237 interactions were supported by AGO/CLASH data covering 539 miRNAs (116 novel miRNA) that targeted 5847 genes.

In this study, we kept two fold change cutoff to identify the up and downregulated miRNAs. Among all, 165 miRNAs were observed upregulated in rhizome tissue at 25 °C whereas 297 miRNA were downregulated. In leaf tissue, 34 miRNAs were upregulated whereas 170 miRNAs were downregulated at 25 °C. Overall 25 °C had lesser number of upregulated miRNAs as compared to that at 15 °C in both the tissues, suggesting a higher requirement of gene regulation at 15 °C.

**Figure 5:**
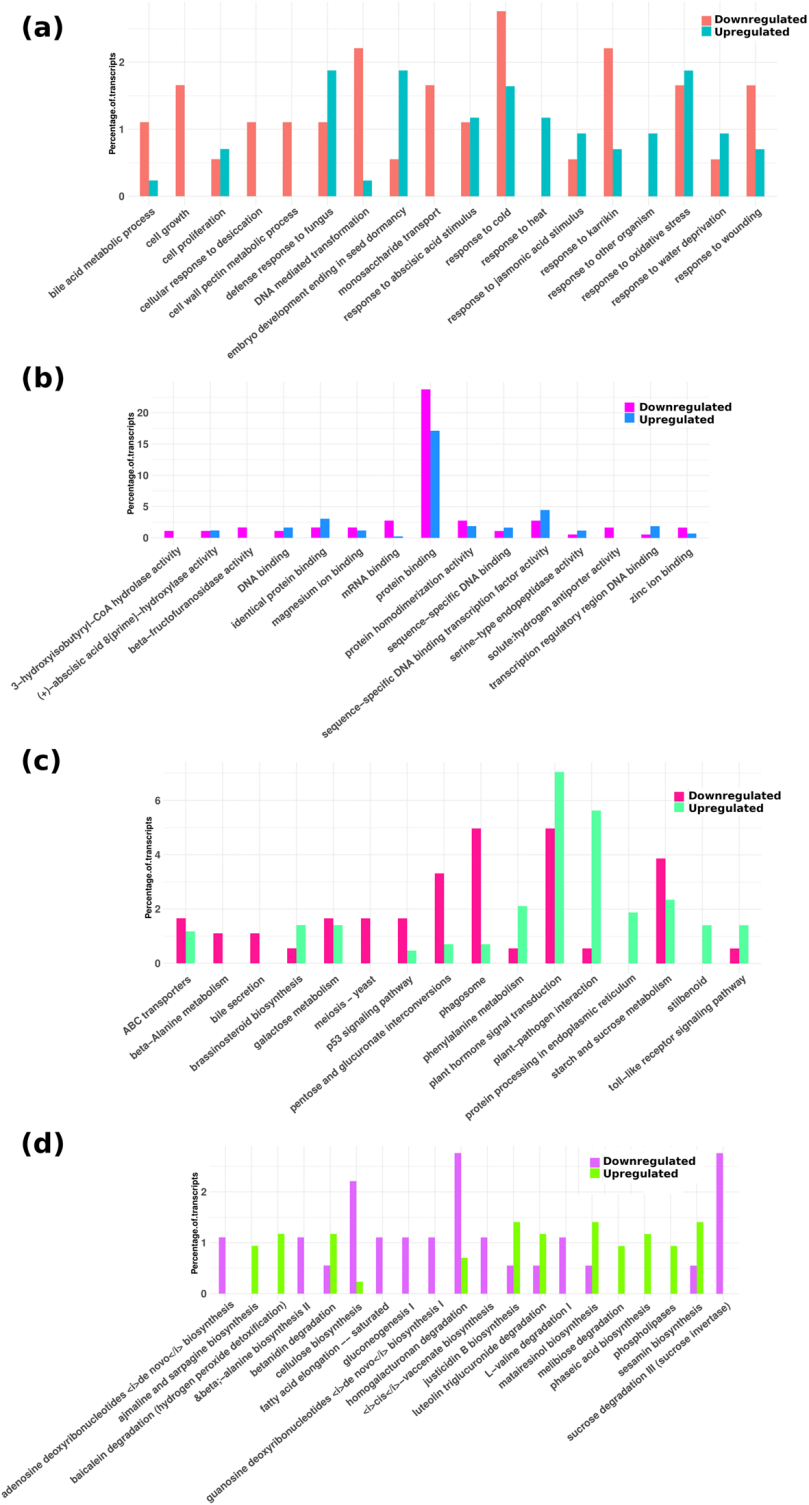
miRNA controlled genes abundance in different categorical annotations in rhizome tissue (**a**) biological process in R25/R15 (**b**) molecular function in R25/R15 (**c**) KEGG pathways in R25/R15 (**d**) PMN pathways in R25/R15.

**Figure 6:**
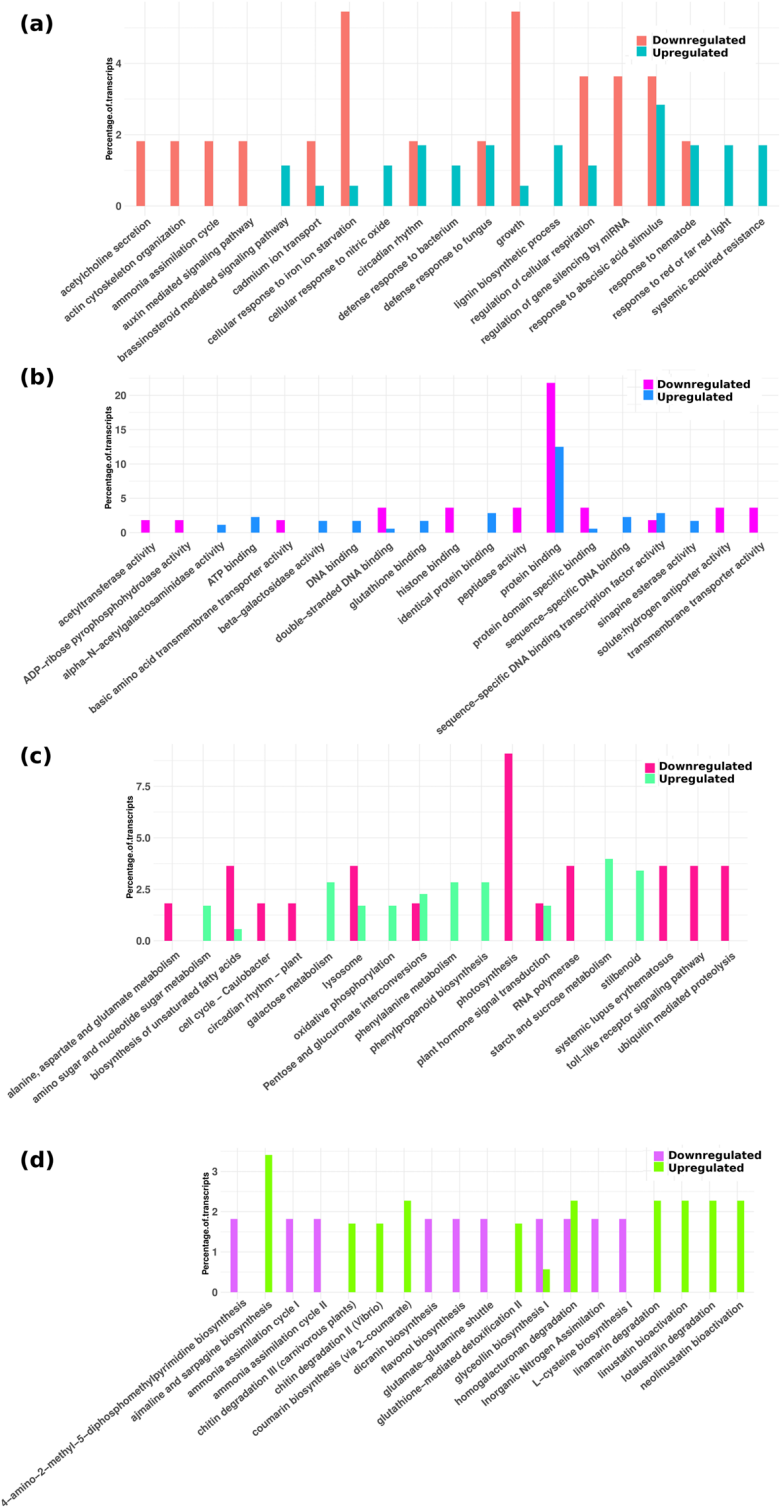
miRNA controlled genes abundance in different categorical annotations in leaf tissue (**a**) biological process in L25/L15 (**b**) molecular function in L25/L15 (**c**) KEGG pathways in L25/L15 (**d**) PMN pathways in L25/L15.

In biological process category, genes belonging to response to cold, response to karrakin, cell growth and monosaccharide transport were downregulated by miRNAs in rhizome tissue at 25 °C as compared to those at 15 °C (Fig. 5a). In leaf tissue, miRNAs downregulated genes associated with growth, regulation of cellular respiration, cellular response to iron ion starvation and actin cytoskeleton organization at 25 °C (Fig. 6a). *P. kurrooa* is a species of high altitude and a higher temperature of 25 °C has been reported to negatively affect its growth and development. The present data suggested a role of miRNAs in affecting slow vegetative growth in *P. kurrooa* at higher temperature. On the contrary, defense response to fungus, response to oxidative stress, response to heat, response to abscisic acid and jasmonic acid stimulus were upregulated, and corresponding miRNAs were downregulated at 25 °C in both the tissues (Figs. 5a, 6a). Under molecular function category, genes involved in sequence-specific DNA binding –transcription factor activity, DNA and protein binding were miRNA controlled and upregulated in both the tissues at 25 °C (Figs. 5b, 6b) indicating a role of miRNAs in temperature mediated modulation of biological processes.

In KEGG pathways, plant hormone signal transduction, starch and sucrose metabolism, galactose metabolism, stilbenoid diarylheptanoid and gingerol biosynthesis, and phenylalanine metabolism pathways were upregulated at 25 °C in both the tissues whereas targeting miRNAs were downregulated (Figs. 5c, 6c). However, plant pathogen interaction, brassinosteriod biosynthesis, and toll like receptor signaling pathways were exclusively upregulated in rhizome tissue exposed to 25 °C (Fig. 5c).

Under PMN pathway category, transcripts belonging to ajmaline and sarpagine biosynthesis, phospholipases, hydrogen peroxide detoxification, glutathione mediated detoxification and homogalacturonan degradation were miRNA controlled and upregulated at 25 °C in both the tissues (Figs. 5d, 6d). Data revealed major metabolism adjustments for physiological and morphological adaptations.

### miRNAs regulate expression of stress responsive genes in *P. kurrooa*

Cytosolic free calcium acts as secondary messenger in abiotic stress signaling. Calmodulins are calcium binding proteins that play an important role in plant stress response by inducing downstream signal transduction pathways^69^. Overexpression of calmodulin like (ShCML44) stress-responsive gene is reported to impart tolerance to multiple abiotic stresses in *Solanum habrochaites*^70^. In our data, calmodulin gene was targeted by pkr-miR-5350d-5p, and upregulated in both rhizome and leaf tissues at 25 °C. Reversible protein phosphorylation is a vital event in signal transduction which regulates several cellular activities in a eukaryotic cell^71^. Phosphorylation and dephosphorylation is mediated by protein kinases and phosphatases respectively, and allows proteins to switch from one state to another rapidly that enables plants to respond to various stress stimuli accurately and rapidly^72^. miRNAs targeting serine threonine protein kinases as well as phosphatases in both the tissues were identified. CBL-interacting serine threonine protein kinase was upregulated at 25 °C in leaf tissue and the targeting miRNAs, pkr-nov-miR-24, pkr-miR-1302, were downregulated. Likewise, in rhizome tissue at 25 °C, expression of serine threonine protein kinase A was upregulated whereas expression of targeting miRNA pkr-miR-4698 was downregulated. Further, pkr-miR-165a-3p targeting protein phosphatases in both the tissues was downregulated at 25 °C. This suggested that plant responds to temperature stress by activation of miRNA regulated downstream signal transduction pathways.

Plant hormones function as key regulators of plant growth, development and complex stress signaling cascades^73^. Abscisic acid (ABA) and ethylene are phytohormones that play an important role in abiotic stress signaling^74,75^. Brassinosteroids are steroidal phytohormones with multifaceted role in plant growth, development and adaptation to abiotic and biotic stresses^76^. In our data, miRNAs were targeting transcripts involved in brassinostreroid, ethylene and abscisic acid mediated signaling pathways in both tissues (Supplementary Fig. S7). Downregulation of miRNAs targeting signaling pathways mediated by these hormones at 25 °C suggested temperature mediated modulation of hormone signaling cascades by miRNAs.

Interestingly, miRNAs targeting transcription factors involved in abiotic stress signaling such as MYB, WRKY and ethylene responsive transcription factors (ERF) were also identified. Since *P. kurrooa* is a medicinal plant species of high altitude where temperature fluctuations are quite high, miRNAs targeting signaling pathways and transcription factors are worth studying in imparting tolerance to temperature stress in *P. kurrooa*.

Reactive oxygen species (ROS) play a dual role in plant stress response by causing oxidative damage and functioning as stress-signalling molecules^77^. Glutathione-S-transferases are antioxidant enzymes that quench ROS with addition of glutathione and thereby protect cells from oxidative damage. Numerous studies suggested a role of glutathione-S-transferases in imparting abiotic stress tolerance to plants^78,79^. For example, over-expression of AtGSTU19 was reported to impart tolerance to salt, drought and methyl viologen stresses in *Arabidopsis thaliana*^80^. In our data, pkr-miR-482c-5p with glutathione-S-transferase as a target in both the tissues was identified. Down regulation of pkr-miR-482c-5p at 25 °C in both the tissues suggested the activation of plant antioxidant machinery to combat oxidative stress caused by ROS.

Temperature is reported to affect cell wall metabolism in plants^81,82^. In our data, enzymes involved in cell wall modifications β-d-xylosidase, β-glucosidase, α-l arabinofuranosidase and polygalactouranase were differentially expressed and the corresponding targeting miRNAs were identified in both the tissues. Overall, up-regulation of stress responsive transcripts and down-regulation of targeting miRNAs at 25 °C suggested a role of miRNAs in regulating plant stress response.

### miRNAs regulate defense related genes in *P. kurrooa*

Temperature has been reported to modulate defense responses in plants^83^. Pathogenesis related (PR) proteins are vital components of plant innate immune system and serve as molecular markers of defense signaling pathways^84^. Several studies have suggested a role of PR proteins in abiotic stress tolerance. Overexpression of PR10 enhanced tolerance to abiotic and biotic stress in rice^85^. Novel miRNAs pkr-nov-miR-352 and pkr-nov-miR-265 with basic form of pathogenesis related protein 1 (PRP1) as target were identified in both the tissues. Further, pkr-miR-7588 was found targeting chitinase, a subgroup of PR proteins in both the tissues. Abiotic stresses have been reported to induce the expression of chitinases in *Brassica juncea*^86^. Overexpression of chitinase from *Trichoderma harzianum* imparted resistance to salinity and heavy metals in transgenic tobacco^87^. An upregulation of PR-related genes at 25 °C suggested miRNA mediated modulation of plant immune response and a cross-talk between abiotic and biotic stress signaling pathways.

**Figure 7:**
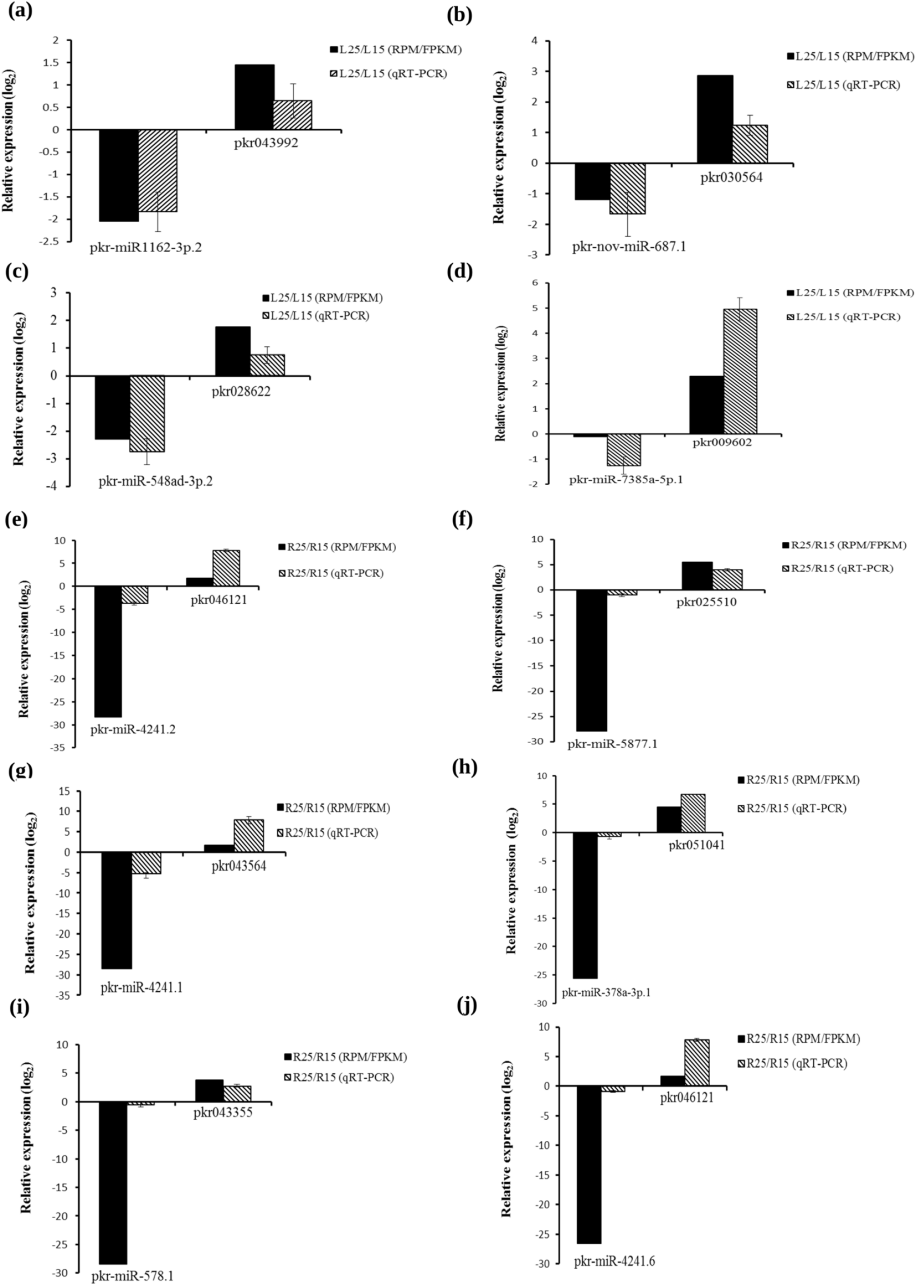
Relative expression of ten miRNAs and respective targets at 25 °C as compared to those at 15 °C based upon data obtained by reads per million (RPM) for miRNA and fragments per kilobase of exon per million fragments values (FPKM) for respective targets followed by validation using qRT-PCR (**a**–**j**). The relative expression values were log_2_ transformed. *Actin* and *5.8 S rRNA* were used as endogenous control to normalize the data for miRNA targets and miRNAs, respectively. Each value in the bar diagram represents mean of three independent biological replicates and error bars represent standard error of mean.

To validate the reliability of above observations, qRT-PCR was used to analyze expression of miRNA and their identified targets. Randomly, 14 pairs of such miRNAs and their identified targets were selected for experimental validation. The expression pattern of 10 out of 14 selected miRNA, and targets pairs obtained by qRT-PCR were consistent with the computational study results. The results revealed significant anti-correlation between expression of miRNAs and corresponding targets (Fig. 7).

Collectively, a total of 462 and 208 differentially expressed temperature responsive miRNAs were identified in *P. kurrooa*. KEGG and PMN-pathway enrichment showed that most of the miRNAs were involved in regulating genes involved in plant stress response, hormone signal transduction and plant pathogen interaction pathways. The study provides a repertoire of temperature responsive miRNAs and corresponding targets which could be used in enhancing stress tolerance in *P. kurrooa*.

### Web-browser based implementation of all the annotation of genome

The draft genome assembly has been implemented as a web server, PicroDB, using HTML5. Jbrowse was used to implement all the tracks of annotation. It is an embeddable genome browser built completely in HTML5 and Javascript. Tabular formats for all the annotation tracks have been provided to make it convenient for users. PicroDB is a comprehensive information database of *P. kurrooa* genome which records data in analytical modes for gene profiling and miRNA binding sites and other regulatory components like repetitive elements, transcription factors and miRNAs. This database is useful to browse the potential regulation of genes analyzing details with miRNAs, repeats and transcription factors. Besides archiving all detailed annotations in highly interactive form, the data and analysis could be accessed in a dynamic way to look up for genes through search, and visually pinpoint the coordinates for their regulatory information. The regulatory search provides all the information at one place including possible transcriptional and post transcriptional factors involved in regulation. Krona plot, radial-tree plot and interactive pie charts were implemented using circles-infographic plots in this database. This highly interactive graph based regulatory system visualization platform has been implemented using advanced graph plotting tools and rich visualization libraries of javascript and D3. It has been made available at https://scbb.ihbt.res.in/picro-db/.

## Conclusion

The assembled draft genome of *P. kurrooa* offers a valuable resource and reference information to understand the biology of this important endangered species. The study also provided a novel computational strategy of assembling, while dealing with highly repetitive complex genome where unique tagging of repeats can resolve complex genomes. The plethora of information releasing from the draft genome of *P. kurrooa* has been archived into an interactive panoramic database which is freely available for the scientific community to take maximum advantage from the findings made here*.* The availability of *P. kurrooa* reference genome will facilitate integration of biotechnological tools for improvement and conservation of this endangered medicinal herb.

## Methods

### Plant material

*P. kurrooa* was collected from natural habitat at Rohtang (32°23′ N, 77°15′ E; 4000 m altitude, India). Indian Biological Diversity Act 2002 permits bonafide Indians to access biological resources for scientific research^88^. Our institute at Palampur (1300 m altitude; 32°06′ N, 76°33′ E, India) has an internationally recognized herbarium with “PLP” as an acronym wherein *P. kurrooa* was deposited as voucher specimen no. 6500. The voucher specimen was identified by the taxonomist at our Institute. At Palampur, *P. kurrooa* plants were transferred in plastic pots containing a mixture of soil, farm yard manure and sand in a ratio of 2:1:1 and maintained for 3 months in the experimental farm of Institute. After 3 months, plants were shifted to a plant growth chamber (Percival Scientific, USA) maintained at 15 °C (16 h of photoperiod; 350 µmol m^−2^ s^−1^ photosynthetic photon flux density).

### Genomic library preparation and sequencing

For genomic library preparation, DNA was isolated using cetyl trimethylammoniumbromide (CTAB) method^89^. The quantity and quality of isolated DNA was checked using a NanoDrop 1000 (NanoDrop Technologies, USA), Agilent 2100 Bioanalyzer (Agilent technologies, USA) and Qubit^®^ 2.0 Fluorometer (Invitrogen, USA). Illumina paired-end libraries were prepared using truseq DNA PCR-free sample prepration kit (Illumina USA) following manufacturer’s instructions. Seven genomic libraries with insert size ranging from 200 to 500 bp were prepared. Paired end sequencing, (2 × 76 bp) and (2 × 101 bp), was carried out on an Illumina Genome Analyzer IIx (Illumina, USA) as per the manufacturer’s instructions. For long read sequencing, genomic libraries were prepared using PacBio 10 Kb and 20 Kb template prep kit (Pacific Biosciences, USA) with Blue Pippin Size Selection System (Sage Science, USA) following manufacturer’s instructions. The libraries were sequenced on the PacBio RS II platform (Pacific Biosciences, USA) using 255 SMRT cells. In total, 80 Gb data (~ 45 × coverage) was generated.

### cDNA and small RNA library preparation and sequencing

*P. kurrooa* plants were kept at 15 °C and 25 °C in two separate plant growth chambers (350 μmol m^−2^ s^−1^ photosynthetic photon flux density; 16 h photoperiod) for 2 weeks followed by sampling of leaf and rhizome tissues at both the temperatures. RNA was isolated as described by Ghawana et al.^90^ and its integrity and concentration was checked by using RNA Nano chip on Agilent Bioanalyzer 2100 (Agilent technologies, USA). cDNA and small RNA libraries were prepared using truseq RNA library preparation kit (Illumina, USA) and truseq small RNA sample preparation kit (Illumina, USA), respectively, following the manufacturer’s instructions. Paired end (PE) 36 × 2 bp sequencing of cDNA libraries was carried out on Illumina Genome Analyzer IIx (Illumina, USA) as per the manufacturer’s instructions. Sequencing of small RNA libraries was also carried out on Illumina Genome Analyzer IIx (Illumina, USA) as per the manufacturer’s instructions.

### Read data filtering

A total of 115 Gb data was generated by sequencing of genomic libraries using Illumina GAIIx sequencing platform. Basecall files from Illumina were converted into fastq file format using bcltofastq.pl script. In order to remove sequencing error, reads were filtered using in-house developed script filteR^8^. This tool was run with two different modules, first one was avg_p which gives position wise quality score for each read file. Therefore, if there is any kind of instrumentation error in any cycle during read generation step on Illumina sequencer then it will be detected on that particular position in each of the read. Whereas, the second module checks for per read quality where adaptor contamination is also checked. If any poor quality read is found, it is discarded. For the first module avg_p, minimum quality score of QV > = 25 for each position was considered. Any read files showing quality value less than 25QV were trimmed from that position. For the second module quality value of 25 for 70% read length were considered. Finally, all high quality reads were used for error correction of PacBio SMRT long reads.

Initial read length for PacBio long reads varied from 36 bp to 65 Kbp. PacBio long reads had ~ 13% error rate which is usually in the form of indels. Therefore, these reads needed to be corrected before using them for assembly. In order to correct these reads, hybrid error correction protocol proovread was used with default parameters. This protocol first of all maps short reads of Illumina across the long reads of PacBio. These mapped files were used for consensus calling for the final corrected reads. Reads with chimeric or low consensus position were trimmed. Along with these hybrid error corrected reads, PacBio self error correction strategy with Canu assembler^18^ was also used.

### Assembly of PacBio long reads

Hybrid error corrected reads and PacBio self corrected reads were used to produce primary assembly draft using Canu assembler with default parameters where 500 bp minimum read length was considered for assembly. Canu assembler assembled contig level assembly which was a lot higher than expected genome size in terms of base pair. Therefore, a novel strategy was developed to assemble these contigs into scaffolds. This strategy is discussed in result section of this article.

### Assembly completeness validation

BUSCO v3.0.2 was downloaded along with (multiple) helping databases including eukaryota_odb9 and viridiplantae_odb10. BUSCO was run using the following command: python scripts/run_BUSCO.py -i picro_assembly.fasta -o picro_embryo -l embryophyta_odb9 -c 30 -m geno and python scripts/run_BUSCO.py -i picro_assembly.fasta -o picro_viridiplantae -l viridiplantae_odb10 -c 30 -m geno. Similarly to run CoreGF, database of CoreGF was download from it’s portal PLAZAv4.0 Eudicotyldone. Respective sequences for Lamiales group of species were retrieved from the database. BLAST was run with these sequences against genome fasta file. Top hits were selected on the basis of E-value-1–05. ESTs were collected from NCBI EST database. BLAST run was performed against the genome to get the tophits for these ESTs.

### Wet lab validation of randomly selected contigs

Primers were designed from 5′ and 3′ ends of 11 supra contigs. The properties of the designed primers were calculated using online oligoCalc tool (http://biotools.nubic.northwestern.edu/OligoCalc.html). Primer sequences and thermal cycling profile used for amplification of 3′ and 5′ end of contigs are provided in Supplementary Table S5. Total DNA was extracted from *P. kurrooa* leaves using CTAB method^89^. For PCR 100 ng/µl DNA was used for amplification of contig sequences using Advantage GC2 Polymerase (Takarabio, Japan).

### Gene annotation

To annotate genes, Core Eukaryotic Genes Mapping Approach (CEGMA) was run across the assembled genome. Output of CEGMA was converted into SNAP HMMs. GeneMark was also run onto genome. HMM from both CEGMA and GeneMark were used into MAKER2's first pass along with evidence sets transcripts from previous transcriptome study. Output of MAKER2's first pass were converted into SNAP HMMs and AUGUSTUS HMMs. MAKER2 was run with more stringent setting along with three HMMs (MAKER SNAP, GeneMark, AUGUSTUS) and evidence set (transcripts). From the final set of genes only Annotation Edit Distance (AED) < 1 were retained.

### Functional and pathway annotation on the basis of homology

Gene sequences along with their most probable transcript and translated protein sequence were retrieved from MAKER2. These transcript sequences were BLAST searched against NCBI NR database to find closest annotation for each transcript. Tophit for each transcript was selected as most probable annotation for that transcript. To functionally classify these transcripts, we used Annot8r tool. Annot8r searches against Uniprot database, Gene Ontology (GO), Kyoto Encyclopedia of Genes and Genomes (KEGG), and Enzyme Commission codes (EC).

### Gene expression measurement

For RPKM (Read Per Kilobase per Million) level measurement of *P. kurrooa* transcripts, reads from four different conditions were mapped back on transcripts of each gene using Bowtie. Number of mapped reads were normalized using RPKM.

### Differential expression analysis

For differential expression (DE) analysis, mapped read counts for each transcript were used according to specific condition. DE was carried out using EdgeR package in R. EdgeR estimated the mean and variance of raw read counts under negative binomial distribution to identify differentially expressed transcripts. P-value < = 0.05 was used to distinguish between significant DEGs and non significant DEGs.

### Plant metabolic network

The Plant Metabolic Network is a broad database of plant metabolic networks for genes, enzymes, compounds, reaction and pathway involved in primary and secondary metabolism in plants. The identified differentially expressed genes were mapped for these networks.

### Experimental validation using quantitative real-time polymerase chain reaction (qRT-PCR)

For expression studies, total RNA was isolated from leaf and rhizome tissue of *P*. *kurrooa* plants exposed to 15 °C and 25 °C using method described by Ghawana et al.^90^. For miRNA target expression analysis, total RNA was treated with RNase-free DNase I (Invitrogen, USA) and was reverse transcribed using Superscript III (Invitrogen, USA) following manufacturer’s instructions. Gene specific expression primers for qRT-PCR were designed using Primer Express 3.0.1 primer design tool (Invitrogen, USA). Primer sequences used for expression analysis are provided in Supplementary Table S6. qRT-PCR was performed with three separate biological replicates using *Actin* as an internal control gene on an Applied Biosystems step one plus real-time PCR system (Applied Biosystems, USA) using 2 × Brilliant II SYBR Green QPCR Master Mix (Agilent Technologies, USA). qRT-PCR was conducted with the following conditions: 10 min at 95 °C, 40 cycles each of 30 s at 95 °C, 60 s at desired Tm followed by a final melting curve analysis (55–95 °C) so as to verify the specificity of amplicons. For miRNA expression analysis, cDNA specific for each miRNA was synthesized using Taqman cDNA synthesis kit following manufacturer’s instructions. Primers and Taqman probes used for miRNA expression analysis were custom synthesized and obtained from Applied Biosystems, USA (Supplementary Table S6). All reactions were performed with three separate biological replicates with *5.8S rRNA* as an internal control for normalization. qRT-PCR was carried out using the StepOne Plus Real-Time PCR System (Applied Biosystems, USA) with the following protocol: 50 °C for 15 min, 95 °C for 10 min (enzyme activation), followed by 40 cycles of denaturing at 95 °C for 15 s, annealing and extension at 60 °C for 1 min. Expression of both targets as well as miRNAs with respective controls was estimated using 2^−ΔΔCT^ method^91^. Expression values were transformed (log_2_) to generate expression profiles.

### miRNA annotation

To annotate miRNAs miRDeep2^92^ scripts were used. Reads obtained by sequencing of small RNA libraries were quality filtered using filteR^8^. After quality assessment, low quality reads along with adaptor contaminated reads were discarded. Final quality reads were collapsed into unique reads with their copy number information. These reads were mapped onto genome using Bowtie. Due to limited number of mismatches (two in seed region), Bowtie was unable to map sufficient number of reads onto genome. Therefore, these reads were mapped onto genome using BLAST and BLAST output was converted into arf file format which is a specific file format for miRDeep2. The arf file, genome file, read files, and known precursors file from mirBase were provided as input into miRDeep2 to find novel and known miRNAs.

### miRNA target sites finding

To assign functions to these miRNAs, target genes were identified using p-TAREF^93^ with maximum of two mismatches using polynomial kernel. Further, pearson correlation coefficient (PCC) was calculated using RPM value of miRNAs and FPKM values of target genes. All those miRNA: target pairs, which showed high negative correlation coefficient (“r” < − 0.5) were selected for further analysis as an experimental evidence in Argonaute protein data from AGO1-9 using Bowtie.

### Transcription factors and their binding sites annotation

Genes annotated from BLAST and Annot8r^59^ were considered as transcription factors if these were showing tophit as a transcription factor from NR database. Other than BLAST hit, transcription factors were also searched on the basis of PFAM domain search. TFBS motifs belonging to 82 Different Plant TF families were downloaded from PlantPAN2.0 database. These matrices were scanned on genes along with their 2 kb upstream regions. To filter out significant binding sites, matrices were mapped to their respective TF family. Transcription factors, belonging to those families and genes on which their binding sites were found, were further checked for correlation =  > 0.8 with significant p-value < 0.05.

### Repeat annotation

RepeatModeler was used to detect and annotate repetitive elements on the genome with de novo repeat family identification and modeling module. This module uses RepeatScout, RECON and Tandem Repeat Finder (TRF) to find repeats in scaffolds using de novo and similarity based approaches. Draft assembly file was provided as an input into RepeatModeler in fasta file format. RepeatModeler constructed a repeat library with de novo and known repeats. This library was fed into the RepeatMasker/Repbase to find all de novo and novel repeats in the primary assembly draft.

### Ribosomal RNA identification

To annotate ribosomal RNA in the *P. kurrooa* genome, RNAmmer 1.2v^65^ standalone version was used. RNAmmer predicts ribosomal RNA on the basis of Hidden Markov Model trained on ribosomal RNA from different kingdoms of life. Genome fasta file was used as the input for RNAmmer. Further predicted ribosomal RNAs were searched for multiple copies in genome using BLASTn.

### Phylogenetic analysis

All transcripts which were found in our data were BLAST searched against NR database of NCBI. Tophits for each transcript were selected on the basis of bitscore and e-value. Unique counts for all species were generated from top hit. For phylogenetic analysis ribosomal RNAs were BLAST searched against nucleotide database of NCBI. The ribosomal RNAs with maximum species number in tophit were selected. The respective ribosomal RNA sequences for different species were retrieved from database and only top 100 hits were retained. These hundred hits were aligned to each other using clustalW. This alignment was converted into Nexus file format which was used as input into BEASTV1.10.4^66^. BEAUti was run with strict molecular clock and GTR substitution model. After running BEAUti it’s xml file was loaded into BEAST to generate tree file. This tree file was loaded into TreeAnnotator to generate MCC tree. Final annotated tree was visualized with FigTree Package. In the similar manner, the phylogenetic analysis was carried out with a chloroplast gene, photosystem II protein D1, whose sequence was available across 74 species found closest to *P. kurrooa* in the BLAST hit. For fourth level analysis, Amino Acid Identity (AAI) profiler^67^ was used. AAI-profiler uses proteome data to taxonomically classify a species. Therefore translated protein sequences from transcripts were used as input for AAI profiler. It takes fasta file of protein sequences as input. Using SANSparallel it searches for each query protein in the Uniprot database. Based on the hit for all query proteins in each species, amino acid identity (AAI) is calculated. If AAI is found higher than 95% then species are considered to be same. AAI generated three kinds of plots: scatterplot, barcharts and krona plot.

### Ethics approval and consent to participate

The study including sample collection was conducted according to India’s Biological Diversity Act 2002 which permits bonafide Indians to use biological resources for scientific research^88^.

## Supplementary Information

Supplementary Information 1.

Supplementary Information 2.

## Data Availability

All the raw data used in this study is submitted to NCBI SRA data repository under the accession number PRJNA660113. All secondary data used in this study are available in supplementary data files provided along with and also made available through the related server at https://scbb.ihbt.res.in/picro-db/.

## Abbreviations

ABA: Abscisic acid
BLAST: Basic local alignment search tool
BUSCO: Benchmarking universal single-copy orthologs
DEG: Differentially expressed genes
EC: Enzyme commission codes
EST: Expressed sequence tag
FPKM: Fragments per kilobase of exon per million
GO: Gene ontology
KEGG: Kyoto encyclopedia of genes and genomes
KOG: Eukaryotic orthologous groups
lincRNA: Long intervening non-coding RNAs
lncRNA: Long non-coding RNAs
NGS: Next-generation sequencing
NR: Non redundancy
ORF: Open reading frame
PacBio: Pacific biosciences
PFAM HMMs: Protein families hidden Markov models
PMN: Plant metabolic network
qRT-PCR: Quantitative real time polymerase chain reaction
RPKM: Reads per kilobase of exon per million mapped reads
SMRT: Single molecule real time sequencing
snoRNA: Small nucleolar RNA
tasiRNA: Trans-acting small interfering RNA
TFDB: Transcription factor database
