# Supplementary material for: The first draft genome of Picrorhiza kurrooa, an endangered medicinal herb from Himalayas
Source: Sci Rep. 2021 Jul 22;11:14944. doi: 10.1038/s41598-021-93495-z (PMC8298464; doi:10.1038/s41598-021-93495-z)
Supplement: Supplementary file 2 — Supplementary Information 2. [file 41598_2021_93495_MOESM2_ESM.pdf]

The first draft genome of *Picrorhiza kurrooa*, an endangered medicinal herb from Himalayas

Tanvi Sharma[1,3†], Nitesh Kumar Sharma[2,3†], Prakash Kumar[2,3], Ganesh Panzade[2,3], Tanuja Rana[1], Mohit Kumar Swarnkar[1], Anil Kumar Singh[1,4], Dharam Singh[1], Ravi Shankar[2,3]*, Sanjay Kumar[1,3]*

Affiliations:

[1]Biotechnology division,

CSIR-Institute of Himalayan Bioresource Technology (CSIR-IHBT),

Palampur (Himachal Pradesh), 176061, India.

[2] Studio of Computational Biology & Bioinformatics,

CSIR-Institute of Himalayan Bioresource Technology (CSIR-IHBT),

Palampur (Himachal Pradesh), 176061, India.

[3]Academy of Scientific and Innovative Research (AcSIR),

Ghaziabad (Uttar Pradesh), 201002, India.

[4] Present address: ICAR-Indian Institute of Agricultural Biotechnology, Ranchi- 834 003, India

[†]Contributed Equally

*Corresponding authors:

Dr. Ravi Shankar, Principal Scientist, CSIR-Institute of Himalayan Bioresource Technology, Palampur (Himachal Pradesh), INDIA, 176061; E-mail: ravish@ihbt.res.in

Dr. Sanjay Kumar, Director, CSIR-Institute of Himalayan Bioresource Technology, Palampur (Himachal Pradesh), INDIA, 176061. Tel: +91 1894-230411, FAX: +91 1894-230433; E-mail: sanjaykumar@ihbt.res.in

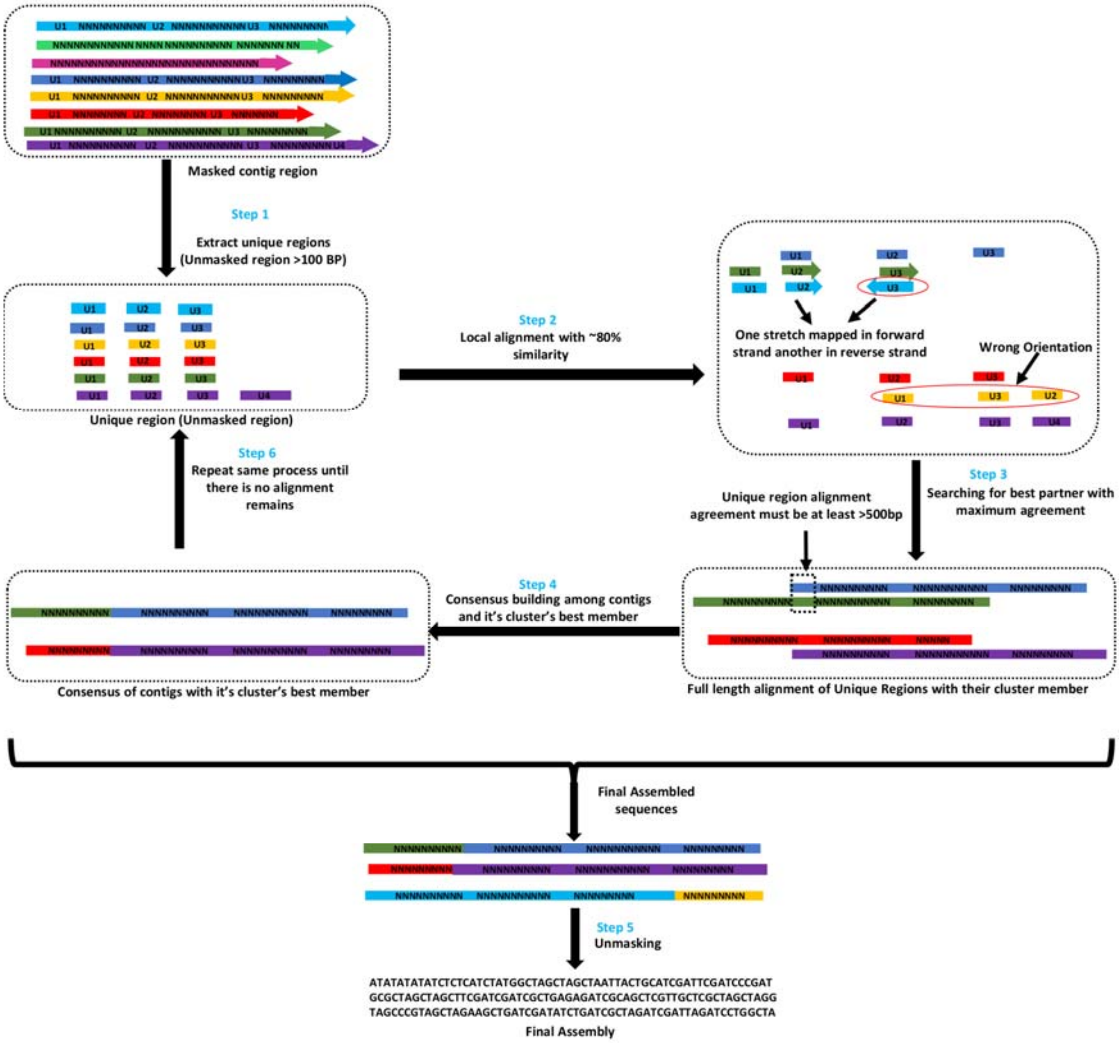

Supplementary Fig. S1 Schematic diagram of novel strategy used to join contigs into scaffold using unique stretches of contigs. In first step, contig unique regions were separated from repeat region. In second step, these unique regions were aligned with each other using BLASTN with 80% similarity. In this step, alignments were checked for three criteria (i) orientation of alignment must be same in all unique stretches of one pair of contigs (ii) order of occurrence of unique region must be same in each pair of contig (iii) length of alignment must be >500 bp to avoid the occurrence of a fragment by chance. In third step, best pairs for each contig were selected. In fourth step, each were merged into a single contig, if best pairs were having more than two contigs then consensus were built. This process was repeated sixteen times and thereafter in fifth step all scaffolds were unmasked.

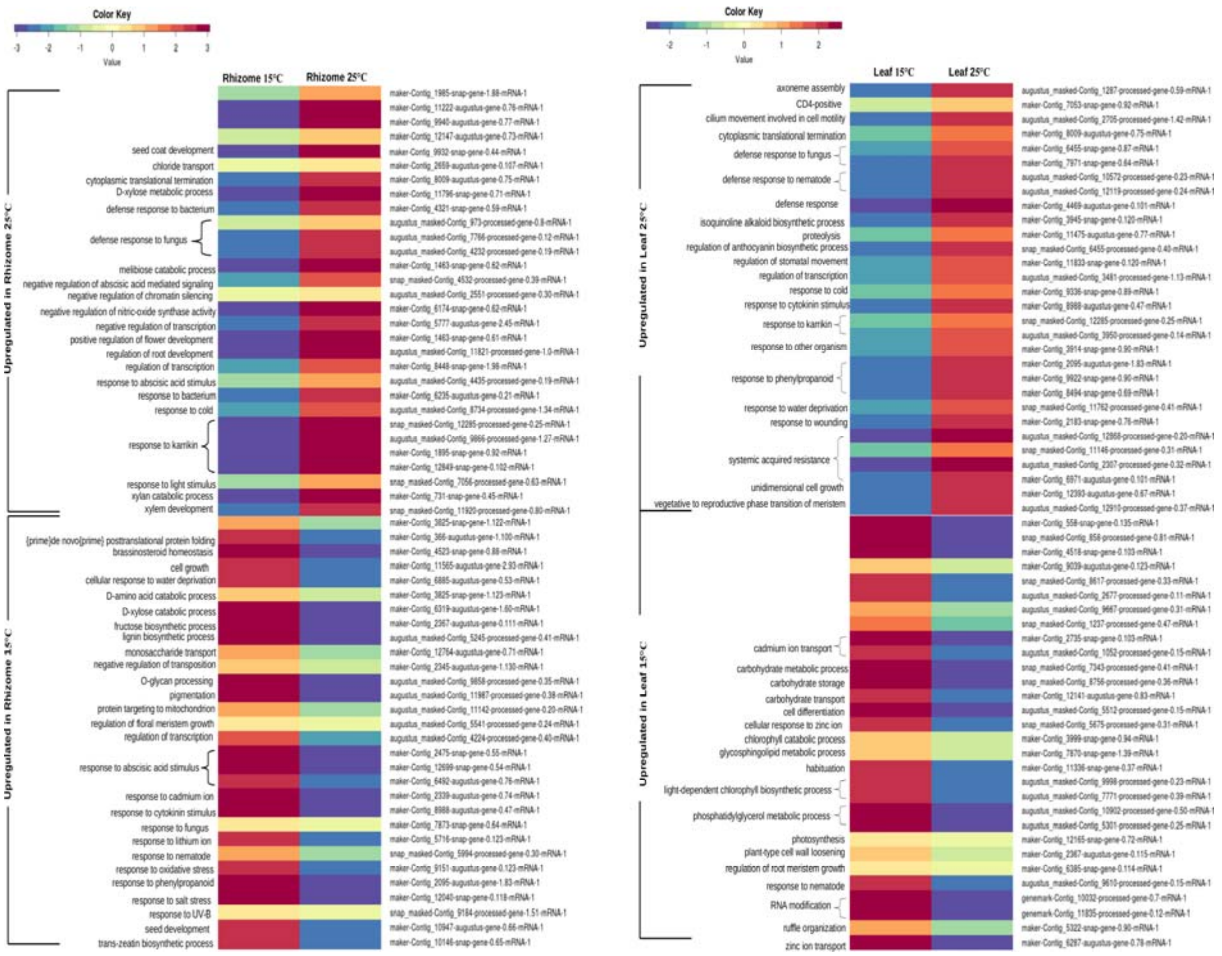

Supplementary Fig. S2 Heatmaps for top 30 differentially expressed transcripts in terms of fold change in four different comparative conditions; 15°C rhizome, 25°C rhizome, 15°C leaf and 25°C leaf.

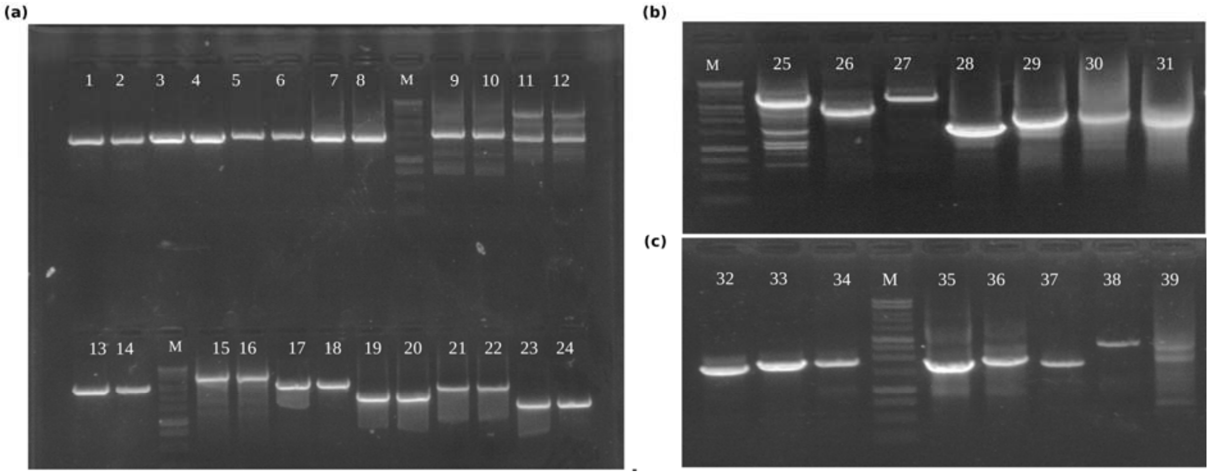

Supplementary Fig. S3 Gel photographs showing amplicons obtained from 3' and 5' ends of respective supra contigs (a,b,c). The well numbers in the gel correspond to superscript on amplicon size in Supplementary Table S1.

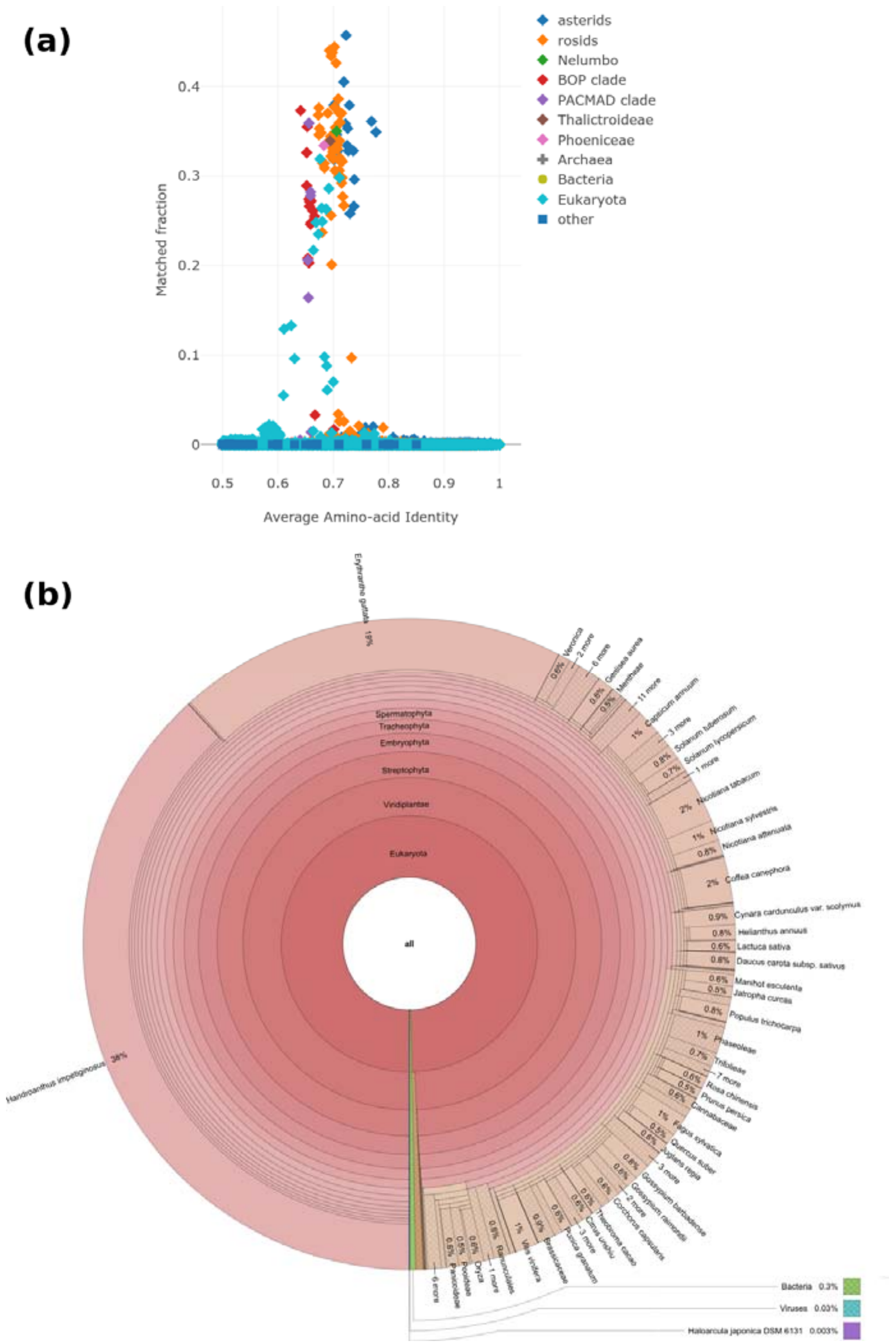

Supplementary Fig. S4 (a) Amino acid identity (AAI) profiling. The plot showed maximum hit with asterids (blue diamond) and rosids (orange diamond). X axis represents the average AAI and Y axis represents the matched fraction. Therefore, top hit species are present at the top-right corner of this plot. **(b)** Krona plot of proteome data of *P. kurrooa*. Each layer of the circle represents different level of taxonomic classification. Outer most layer represents uniprot database hit for *P. kurrooa* data from different species.

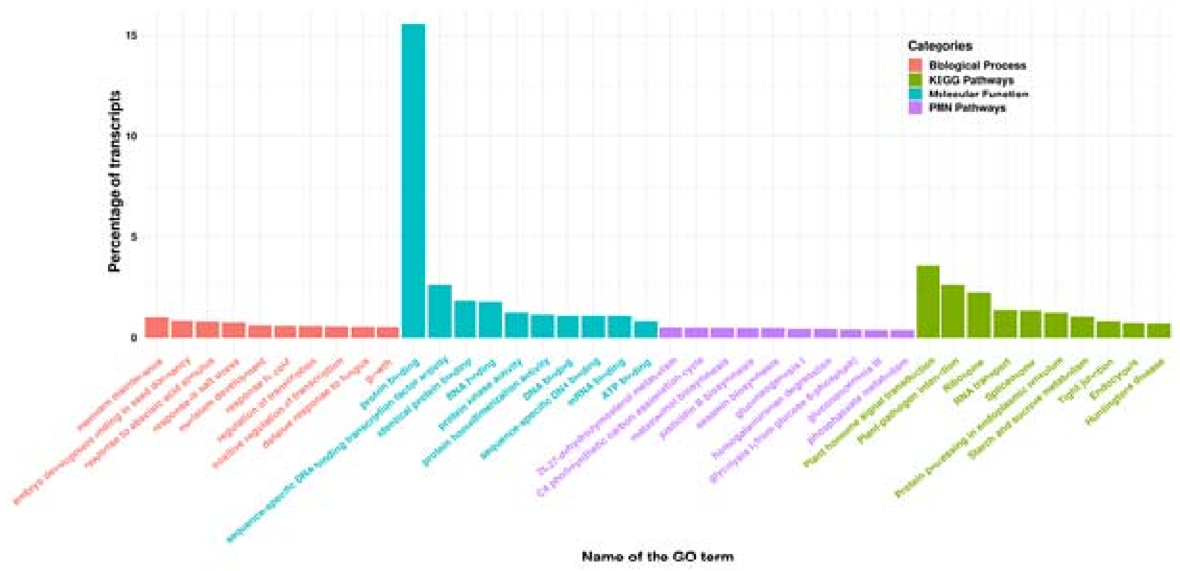

Supplementary Fig. S5 Top abundance of overall assembled transcripts in different categorical annotations; biological process, molecular functions, PMN pathways and KEGG pathways.

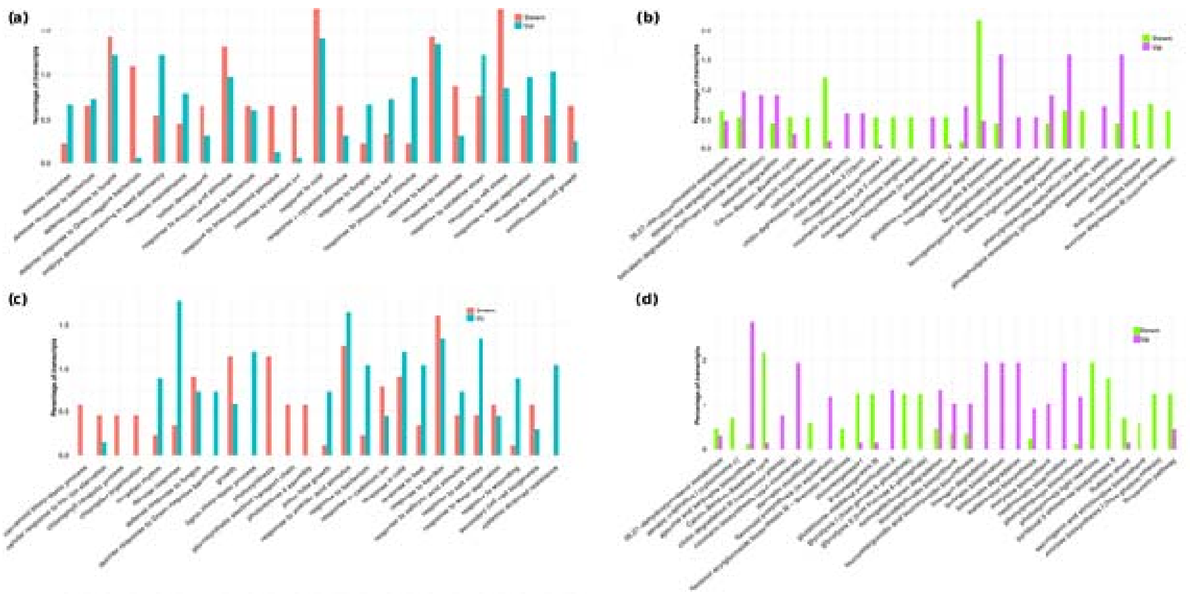

Supplementary Fig. S6 Top abundance of differentially expressed transcripts in rhizome (R25/R15) and leaf (L25/L15) tissue under different categorical annotations (a), (c) biological process (b), (d) PMN pathways.

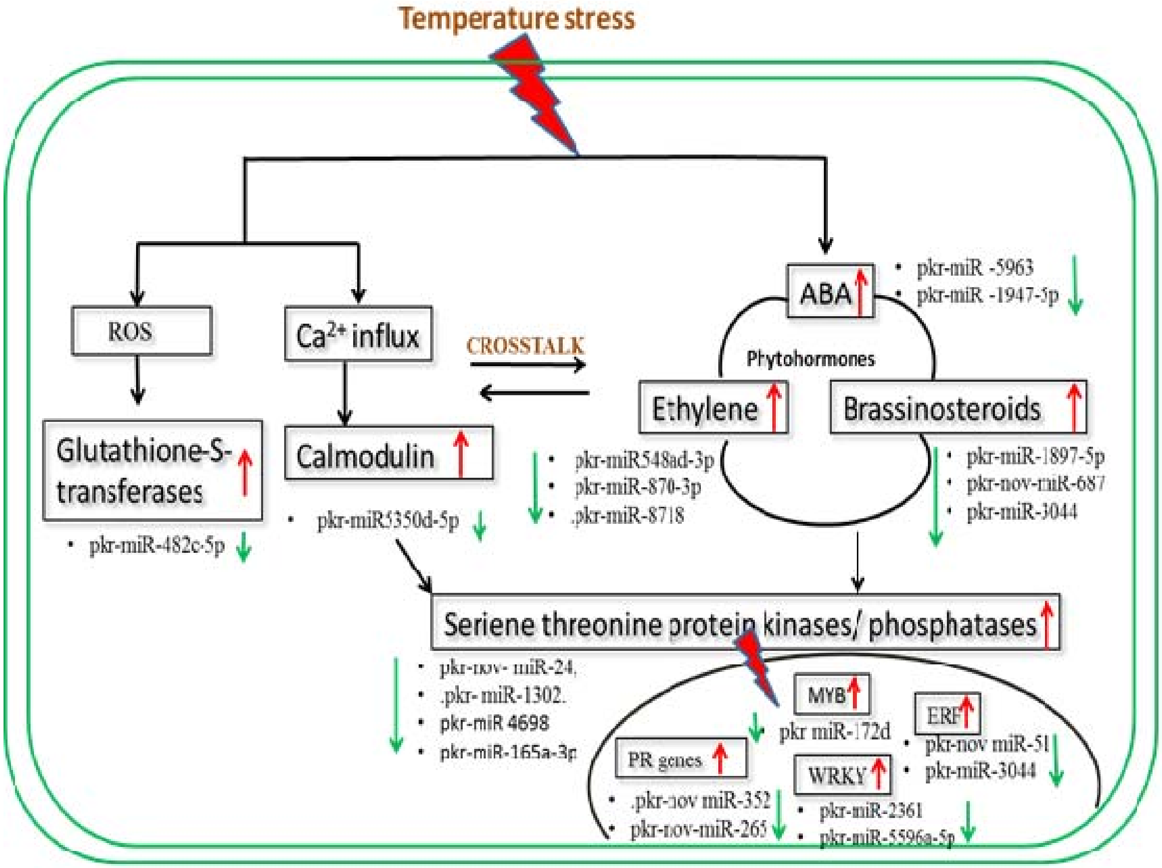

Supplementary Fig. S7 A proposed model depicting temperature stress response regulated by miRNA in *P. kurrooa*. A temperature of 25°C acts as a stress signal which leads to activation of downstream signaling cascades mediated by calcium, ROS and phytohormones including abscisic acid, ethylene and brassinosteroids. The downstream activation of protein kinases and phosphatases subsequently leads to activation of WRKY, MYB and ERF family of TFs as well as PR genes in *P. kurrooa*. The upregulated target genes and corresponding downregulated miRNAs are represented by red and green arrows, respectively.

Supplementary Table S1 Table showing supra contig numbers, expected amplicon sizes, contig end amplified and standardized annealing temperature. The superscript on amplicon size corresponds to the well number shown in the gel in Supplementary figure S3.

**S. No**	**Contig Number**	**Expected PCR amplicon size in base pairs (bp)**	**Contig end amplified (5'end/3'end)**	**Annealing temperature (Ta in °C) with Advantage GC2 Polymerase (Takara)**
**1.**	Contig_3840	1706 bp[1,2]	5'end	60
2.	Contig_5857	1876 bp[3,4]	3'end	60
3.	Contig_8777	1776 bp[5,6]	5'end	60
4.	Contig_2896	1812 bp[7,8]	5'end	60
5.	Contig_6284	2002 bp[9,10]	5'end	60
6.	Contig_6284	1811 bp[11,12]	3'end	60
7.	Contig_12645	2815 bp[13,14]	5'end	60
8.	Contig_115	4745 bp[15,16]	5'end	60
		3278 bp[17,18]	5'end	60
9.	Contig_4012	2734 bp[19,20]	5'end	60
		2210 bp[21,22]	3'end	55
10.	Contig_115	1599 bp[23,24]	3'end	52
11.	Contig_12645	2913 bp[25]	3'end	68
12.	Contig_5857	2824 bp[26]	5'end	60
13.	Contig_115	4666 bp[27]	5'end	55
14.	Contig_9572	1533 bp[28]	3'end	52
15.	Contig_8777	2102 bp[29]	3'end	60
16.	Contig_9572	2196 bp[30]	5'end	60
17.	Contig_3241	1882 bp[31]	5'end	60
18.	Contig_8754	1576 bp[32]	3'end	55
		1748 bp[33, 37]	5'end	55
19.	Contig_4012	1874 bp[34, 36]	5'end	55
20.	Contig_3241	1556 bp[35]	3'end	55
21.	Contig_2896	2760 bp[38]	5'end	55
22.	Contig_2896	2445 bp[39]	3'end	60

Supplementary Table S2 Overall distribution all repeat families found in *P. kurrooa* genome. Novel repeats were around 23.61% whereas in known repeat family, maximum abundant family found was LTR including Gypsy (22.54%) and Copia (20.96%).

**Total number of base pair**	**Repe at family name**	**Percentage distribution**
371206457	Unknown	23.6101479108
354375073	LTR/Gypsy	22.5396076271
329581858	LTR/Copia	20.962664494
42861419	DNA/CMC-EnSpm	2.7261498909
19069998	LINE/L1	1.212924681
17170706	DNA/MULE-MuDR	1.0921224584
11068034	DNA/hAT-Ac	0.7039692195
9305922	RC/Helitron	0.5918921687
8538526	DNA/hAT-Tag1	0.5430828532
7084318	Simple_repeat	0.4505896723
6248600	LTR/Cassandra	0.3974348168
5430268	DNA/TcMar-Stowaway	0.3453857773
3302100	LTR/Caulimovirus	0.2100261672
2017782	DNA/hAT-Tip100	0.128338639
1529713	Low_complexity	0.0972955872
1524784	DNA/PIF-Harbinger	0.0969820839
1074262	SINE/tRNA	0.0683271647
780977	LINE/CR1	0.0496731189
751191	rRNA	0.0477786156
685342	LINE/RTE-X	0.0435903678
456086	LINE/L2	0.0290088109
419032	DNA/hAT	0.026652035

383165	LTR/Pao	0.0243707569
337786	DNA/TcMar-Pogo	0.0214844792
156427	LINE/RTE-BovB	0.0099493544
135870	SINE	0.0086418507
131508	LINE/R1	0.0083644109
111293	DNA/Dada	0.0070786597
103626	LINE/Tad1	0.0065910092
30571	LTR/ERV4	0.0019444323

Supplementary Table S3 Microsatellite marker classification on the basis of repeat unit and most prevalent microsatellite markers in each category. In maximum abundance, monomer unit of repeats were found. In monomer unit A and T nucleotide were occurring most frequently.

Unit size	Number of SSRs	Four top most microsatellite in each category			
1	171303	A=70263	C=15046	G=15041	T=70953
2	59279	TA=21525	AT=19627	TG=2730	AG=2675
3	25009	AAT=2075	TCT=1965	ATT=1926	AGA=1510
4	1554	ATAG=87	ATCT=69	TTAT=65	AAAT=55
5	860	TTCGC=135	CGAAG=130	TTTTA=42	CGCGA=23
6	403	TCGTCA=20	GGGCTT=19	ACGATG=18	TCGTAT=16

Supplementary Table S4 Distribution of top 10 different species which were found most abundant from transcript BLAST tophit.

Name of species	Number of transcript found in a species (%)
Sesamum indicum	8901 (36.31%)
Handroanthus impetiginosus	4447 (18.14%)
Erythranthe guttata	2186 (8.92%)
Olea europaea var. sylvestris	1184 (4.83%)
Dorcoceras hygrometricum	767 (3.13%)
Beta vulgaris subsp. vulgaris	534 (2.18%)
Ipomoea nil	435 (1.77%)
Chenopodium quinoa	274 (1.12%)
Helianthus annuus	126 (0.51%)
Cauliflower mosaic virus	119 (0.49%)

Supplementary Table S5 Primer sequences and PCR thermal cycling profile used for amplification of 3' and 5' end of contigs.

**Primer Name**	**Sequence 5'-3'**	**PCR thermal cycling profile**
Contig_115Fwd1	ATTCTATAAAGTAAGGTAAGGATGATT	94°C for 4 min; 94°C for 30 sec, 60°C for 30 sec, 72°C for 3 min, 30 cycles; 72°C for 5min
Contig_115Rev1	AGTGATTTCAAGCCCTCTGCG	
Contig_115Fwd2	CGCAGAGGGCTTGAAATCACT	94°C for 4 min; 94°C for 30 sec, 60°C for 30 sec, 72°C for 1 min 20 sec, 30 cycles; 72°C for 5min.
Contig_115Rev2	CTTTCTTTCCTTAACGACATCTCA	
Contig_115Fwd3	TGAGATGTCGTTAAGGAAAGAAAG	94°C for 4 min; 94°C for 30 sec, 55°C for 30 sec, 72°C for 1 min, 30 cycles; 72°C for 5min.
Contig_115Rev3	CTACGAAAAAAAAAAAAAAGAGAAAAGAGA	
Contig_115Fwd4	CGAAGAGCATTCACTTTCCATTG	94°C for 4 min; 94°C for 30 sec, 52°C for 30 sec, 72°C for 3 min, 30 cycles; 72°C for 5min.
Contig_115Rev4	TGTGGTTGGGAACATGGACC	
Contig_12645Fwd1	CCATAGCTCGTTTCCCATAATAC	94°C for 4 min; 94°C for 30 sec, 60°C for 30 sec, 72°C for 3 min, 30 cycles; 72°C for
Contig_12645Rev1	TTGCACAAGTTTCTTCACAATTCC	

		5min.
Contig_12645Fwd2	AGCGTGGCTCGAACAGGC	94°C for 4 min; 94°C for 30 sec, 68°C for 30 sec, 72°C for 3 min, 30 cycles; 72°C for 5min.
Contig_12645Rev2	TAGTGACGGAAATATCCGTCAC	
Contig_4012Fwd1	ATTCTTTAGGCTTGGAGCCCTT	94°C for 4 min; 94°C for 30 sec, 60°C for 30 sec, 72°C for 3 min, 30 cycles; 72°C for 5min.
Contig_4012Rev1	AGACATATACAAATAGTTTATCGGTAG	
Contig_4012Fwd2	CTACCGATAAACTATTTGTATATGTCT	94°C for 4 min; 94°C for 30 sec, 55°C for 30 sec, 72°C for 3 min, 30 cycles; 72°C for 5min.
Contig_4012Rev2	TTCTTAAGGAACACATACTCACAC	
Contig_8777Fwd1	GTTTTCCTTTTTTTACAGTGAGACC	94°C for 4 min; 94°C for 30 sec, 60°C for 30 sec, 72°C for 3 min, 30 cycles; 72°C for 5min.
Contig_8777Rev1	GGGTGATAGCATTGATAAAGGAAT	
Contig_8777Fwd2	CTCATACTAGAGTTGGAAGCTG	94°C for 4 min; 94°C for 30 sec, 60°C for 30 sec, 72°C for 3 min, 30 cycles; 72°C for 5min.
Contig_8777Rev2	CCAAACGACAATCCCAAACAGAA	
Contig_5857Fwd1	GTTAGAGAGGAAAGGATTCCTAG	94°C for 4 min; 94°C for 30 sec, 60°C for 30 sec,
Contig_5857Rev1	ACTGTGATTTTCATGCTTCCGCA	

		72°C for 3 min, 30 cycles; 72°C for 5min.
Contig_5857Fwd 2	GTGGCTCCTTTGATTGATTCGT	94°C for 4 min; 94°C for 30 sec, 60°C for 30 sec, 72°C for 3 min, 30 cycles; 72°C for 5min.
Contig_5857Rev2	GAATCAAAGTATGACGAGTGCTC	
Contig_2896Fwd1	GATCTAGTGTTTCTCCAGGTTTC	94°C for 4 min; 94°C for 30 sec, 55°C for 30 sec, 72°C for 3 min, 30 cycles; 72°C for 5min.
Contig_2896Rev1	GAGTTGCATTGAAGTTCGCATC	
Contig_2896Fwd2	GATACGAGCAGTCATCAAGTCT	94°C for 4 min; 94°C for 30 sec, 60°C for 30 sec, 72°C for 3 min, 30 cycles; 72°C for 5min.
Contig_2896Rev2	ACCTAGCTAAGAGAGTGACTG	
Contig_2896Fwd3	AAGTCTCTCATATCTGCGCCAA	94°C for 4 min; 94°C for 30 sec, 60°C for 30 sec, 72°C for 3 min, 30 cycles; 72°C for 5min.
Contig_2896Rev3	CAGACAGACCTCAGAGAGATATT	
Contig_9572Fwd1	TTGATTCCCCTCTCATTCACTAC	94°C for 4 min; 94°C for 30 sec,

Contig_9572Rev1	AGATATGTCCAATAGTTTTGAATGTTTGA	60°C for 30 sec, 72°C for 3 min, 30 cycles; 72°C for 5min.
Contig_9572Fwd2	ATGAACTGCTGACAAAATAGAAATAAAA	94°C for 4 min; 94°C for 30 sec, 52°C for 30 sec, 72°C for 3 min, 30 cycles; 72°C for 5min.
Contig_9572Rev2	AGAGTTGTTAAGTCTCGAGAGGAA	
Contig_3241Fwd1	GCCTTCCAGTGTTCATTTTCAG	94°C for 4 min; 94°C for 30 sec, 60°C for 30 sec, 72°C for 3 min, 30 cycles; 72°C for 5min.
Contig_3241Rev1	CAAGTACTTTGTGACTTTCATTGATG	
Contig_3241Fwd2	AATTTCATTTTTTCGAGTTTTACATCATG	94°C for 4 min; 94°C for 30 sec, 60°C for 30 sec, 72°C for 3 min, 30 cycles; 72°C for 5min.
Contig_3241Rev2	CATGATGTAAAACTCGAAAAAATGAAATT	
Contig_6284 Fwd1	GATGCTGCTGCTGAAAAGGC	94°C for 4 min; 94°C for 30 sec, 60°C for 30 sec, 72°C for 3 min, 30 cycles; 72°C for 5min.
Contig_6284 Rev1	TAAATATGCGTGTATCAAAAGTGTGT	
Contig_6284 Fwd2	TGCCTGATGCGATCTCCTGT	94°C for 4 min; 94°C for 30 sec,

Contig_6284 Rev2	CCCTTAAATTTGAAGACCCAAGAA	60°C for 30 sec, 72°C for 3 min, 30 cycles; 72°C for 5min.
Contig_8754 Fwd1	CGTGCGTACGACACATTTGG	94°C for 4 min; 94°C for 30 sec, 55°C for 30 sec, 72°C for 3 min, 30 cycles; 72°C for 5min.
Contig_8754 Rev1	ACGGTTCACACACTTTACTATTCA	
Contig_8754 Fwd2	TTCCAATTACCTTATCACCTGAATG	94°C for 4 min; 94°C for 30 sec, 55°C for 30 sec, 72°C for 3 min, 30 cycles; 72°C for 5min
Contig_8754 Rev2	CAAGAGCTATATTCTTCTCCAATTTGAT A	
Contig_3840 Fwd1	GACCCTATTCTTATCCGAAGTG	94°C for 4 min; 94°C for 30 sec, 60°C for 30 sec, 72°C for 3 min, 30 cycles; 72°C for 5min.
Contig_3840 Rev1	TCTTCAATGCCCACATCCATGA	

Supplementary Table S6 Primer sequences used for expression analysis of miRNA targets and miRNAs.

**Primers used for expression analysis of miRNA targets**		
**Prmer Name**	**Sequence 5'-3'**	**PCR thermal cycling profile**
pkr043992 Fwd	CGGGAATTACAACGCAAAGTTATC	Initial denaturation at 94° C for 3 min,

pkr043992 Rev	TGGGCATGTTATATGGGATTCTC	followed by 30 cycles of 94° C, 30 sec; 58° C, 30 sec; 72° C, 2 min. Final extension at 72° C for 5 min
pkr030564 Fwd	GCCCGTGGATTTTCGAAA	Initial denaturation at 94° C for 3 min, followed by 30 cycles of 94° C, 30 sec; 58° C, 30 sec; 72° C, 2 min. Final extension at 72° C for 5 min
pkr030564 Rev	TTTCTTTGGCGCGATTAGGAT	
pkr028622 Fwd	CGAGAATTGGCAGAAGCAACTCG	Initial denaturation at 94° C for 3 min, followed by 30 cycles of 94° C, 30 sec; 58° C, 30 sec; 72° C, 2 min. Final extension at 72° C for 5 min
pkr028622 Rev	AAGTGGTTCGAGGCAGCAGT	
pkr009602 Fwd	GGTGTGGAGCGGGTGGAG	Initial denaturation at 94° C for 3 min, followed by 30 cycles of 94° C, 30 sec; 58° C, 30 sec; 72° C, 2 min. Final extension at 72° C for 5 min
pkr009602 Rev	AGAAGCTGCCCGGTGGTC	
pkr046121 Fwd	CCGCCACACATGAACATCAG	Initial denaturation at 94° C for 3 min, followed by 30 cycles of 94° C, 30 sec; 58° C, 30 sec; 72° C, 2 min. Final extension at 72° C for 5 min
pkr046121 Rev	CCGCGAGGAAGGAAAACTG	

pkr025510 Fwd	GGCCATTTGAGAAACCGATTT	Initial denaturation at 94° C for 3 min, followed by 30 cycles of 94° C, 30 sec; 58° C, 30 sec; 72° C, 2 min. Final extension at 72° C for 5 min
pkr025510 Rev	GATTGATCACCATTCCGTCAACT	
pkr043564 Fwd	CCAGTCGCCGCTACAACAC	Initial denaturation at 94° C for 3 min, followed by 30 cycles of 94° C, 30 sec; 58° C, 30 sec; 72° C, 2 min. Final extension at 72° C for 5 min
pkr043564 Rev	CGAACACCTGAACTTCCAAATAGA	
pkr051041 Fwd	GTTAAGAGCTTGTCGCCGAAA	Initial denaturation at 94° C for 3 min, followed by 30 cycles of 94° C, 30 sec; 58° C, 30 sec; 72° C, 2 min. Final extension at 72° C for 5 min
pkr051041 Rev	CGTCTCACAAAACCGTTGAAAA	
pkr043355 Fwd	CGACGTCATCCTCCTTTCAGA	Initial denaturation at 94° C for 3 min, followed by 30 cycles of 94° C, 30 sec; 58° C, 30 sec; 72° C, 2 min. Final extension at 72° C for 5 min
pkr043355 Rev	GGCGACGCCCTTTCTTTC	
pkActin Fwd	GGCTGGAAGAGCACCTCAGGG	94° C for 3 min, followed by 30 cycles of 94° C, 30 sec; 59° C, 30 sec; 72° C, 2 min. Final extension at 72° C for 5 min
pkActin Rev	CGTCTAAGACCAACTCGGCCGTC	

Primers used for expression analysis of miRNAs		
**miRNA**	**Sequence**	**Taqman Assay ID (Applied Biosystems)**
pkr-miR1162-3p.2	AGTTGTAGGCTGTTGAAGAAGATC	CTAAAFC
pkr-miR-7385a-5p.1	TGGTCGGACTGCCTGAGGTCAGTC	CTTZ9CD
pkr-nov-miR-687.1	TTACATAGGATTGGCACGAAGC	CTMFWZN
pkr-miR-548ad-3p.2	AAAAACGAGAACCGAACACCGAAC	CTPRJ6H
pkr-miR4241.2	ATTTGGGAATGACGATTTAACACT	CTXGPZA
pkr-miR-5877.1	ATCGTATCCGAACTTTGTTGGCCC	CTZTD6Y
pkr-miR4241.1	ATTTGGGAATAACGCTTTAACACT	CT2W7RW
pkr-miR-378a-3p.1	ACTGGACTGGGTCTGAGTCGGACC	CT322CU
pkr-miR-578.1	ATTCTTGTGTTAGACTGTTTAACG .	CT7DPGN
pkr-miR4241.6	ATTTGGGAATGACGTTTTAACACT	CT9HH2K
pkr 5.8s rRNA	CAACGGATATCTCGGCTCTC	CTCE3Y9

Filtering of reads

A total of 4,314,919,008 reads were subjected to average quality check. The 3' end of reads having QV<30 were prone to sequencing error. Out of 302 read files, 34 read files were subjected to trimming of reads with varying base pair length due to lower sequencing quality towards the 3' end. For the next step of quality filtering, each read was checked for adaptor contamination or low quality reads.
